# Polymorphs of Nb_2_O_5_ Compound and Their Electrical Energy Storage Applications

**DOI:** 10.3390/ma16216956

**Published:** 2023-10-30

**Authors:** Rui Pang, Zhiqiang Wang, Jinkai Li, Kunfeng Chen

**Affiliations:** 1 School of Material Science and Engineering, University of Jinan, Jinan 250022, China; p15688864578@163.com; 2 State Key Laboratory of Crystal Materials, Institute of Novel Semiconductors, Shandong University, Jinan 250100, China; wangzhiqiang@mail.sdu.edu.cn

**Keywords:** niobium oxide, crystal phase synthesis, electrode materials, electrical properties

## Abstract

Niobium pentoxide (Nb_2_O_5_), as an important dielectric and semiconductor material, has numerous crystal polymorphs, higher chemical stability than water and oxygen, and a higher melt point than most metal oxides. Nb_2_O_5_ materials have been extensively studied in electrochemistry, lithium batteries, catalysts, ionic liquid gating, and microelectronics. Nb_2_O_5_ polymorphs provide a model system for studying structure–property relationships. For example, the T-Nb_2_O_5_ polymorph has two-dimensional layers with very low steric hindrance, allowing for rapid Li-ion migration. With the ever-increasing energy crisis, the excellent electrical properties of Nb_2_O_5_ polymorphs have made them a research hotspot for potential applications in lithium-ion batteries (LIBs) and supercapacitors (SCs). The basic properties, crystal structures, synthesis methods, and applications of Nb_2_O_5_ polymorphs are reviewed in this article. Future research directions related to this material are also briefly discussed.

## 1. Introduction

**Figure 1:**
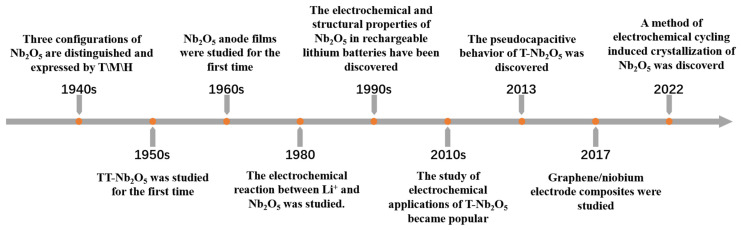
Milestone events of Nb_2_O_5_ research.

Recently, enthusiasm surrounding research on the structure, properties, and applications of Nb_2_O_5_ has grown. In particular, its potential use as catalysts, photocatalysts, supercapacitors (SCs), lithium-ion batteries (LIBs), etc., has been investigated. In nature, niobium (Nb) does not occur in a free state but rather is typically found in a mineral form, (Fe, Mn)Nb_2_O_6_, known as columbite [1,2,3]. Research on Nb_2_O_5_ commenced in the 1940s, with its crystal form the first to be evaluated [4]. The historical research progress on Nb_2_O_5_ is shown in Figure 1. Nb_2_O_5_ exhibits plentiful polymorphs linked to NbO_6_ or NbO_7_ polyhedra by corner- or edge-sharing forms, leading to exceptional properties and various applications [1,2]. As a white powder, Nb_2_O_5_ exhibits redox properties and is non-toxic and insoluble in water and acid but soluble in molten potassium bisulfate, alkali metal carbonates, and hydroxides [5].

Besides its structure, Nb_2_O_5_ has garnered significant attention due to its morphology, size, and corresponding properties. The nanostructured Nb_2_O_5_ often has distinct physical and chemical properties compared to its bulk forms due to its quantum effect and high specific surface area ratio [6]. Nb_2_O_5_ nanostructures and thin films have undergone thorough investigation as promising electrode materials for LIBs and SCs. Nb_2_O_5_ has charge/discharge plateaus (1–2 V vs. Li^0^/Li^+^) and two redox couples (Nb^5+^/Nb^4+^ and Nb^4+^/Nb^3+^), resulting in higher specific capacities [7]. Orthorhombic Nb_2_O_5_ possesses a two-dimensional Li-ion transport pathway without kinetics limitations, facilitating a rapid charge rate [8]. As such, research has focused considerably on controlling the crystal phase, nanostructured Nb_2_O_5_ synthesis, and composite formation [9].

This article provides a comprehensive overview of Nb_2_O_5_, encompassing its crystal structure, preparation techniques, fundamental properties, and diverse applications. It places a particular emphasis on the in-depth exploration of Nb_2_O_5_’s usage in LIBs and SCs. The final section discusses the potential for further research on Nb_2_O_5_ to expand its application scope. 

## 2. Polymorphs of Nb_2_O_5_ and Synthesis Methods

The subsequent section provides a detailed description of the fundamental properties of Nb_2_O_5_, encompassing its synthesis methods, crystal structure, and product morphology. An overview of the physical and chemical properties of Nb_2_O_5_ is also provided, covering the fundamental aspects of this material.

### 2.1. Polymorphs of Nb_2_O_5_

Nb_2_O_5_ is a white crystal or powder with a wide band gap that boasts a relative density of 4.6 and an exceptionally high melting point of 1520 °C. Upon heating, it becomes yellow and exhibits solubility in hydrofluoric acid and sulfuric acid while remaining insoluble in water. Nb_2_O_5_ has diverse physical and chemical properties, making it highly valuable in various applications. Meanwhile, it remains stable in air, insoluble in water, complex in structure, and exhibits extensive polymorphism [1]. In recent decades, researchers have identified more than 15 distinct crystalline phases of Nb_2_O_5_ (as shown in Table 1), including TT-, T-, B-, M-, N-, P-, R-, and H-Nb_2_O_5_. These diverse configurations have unique properties and can be utilized in a wide range of applications [10].

However, not all crystal phases of Nb_2_O_5_ are equally prevalent. The most common include TT-Nb_2_O_5_, T-Nb_2_O_5_, and H-Nb_2_O_5_; their corresponding lattice parameters are shown in Table 2. These crystal structures possess distinct characteristics and can be utilized in various applications [1,19]. Notably, in certain articles, the crystal phases of Nb_2_O_5_ have been re-named as γ (i.e., T), β (i.e., M), and α (i.e., H) [1].

**Figure 2:**
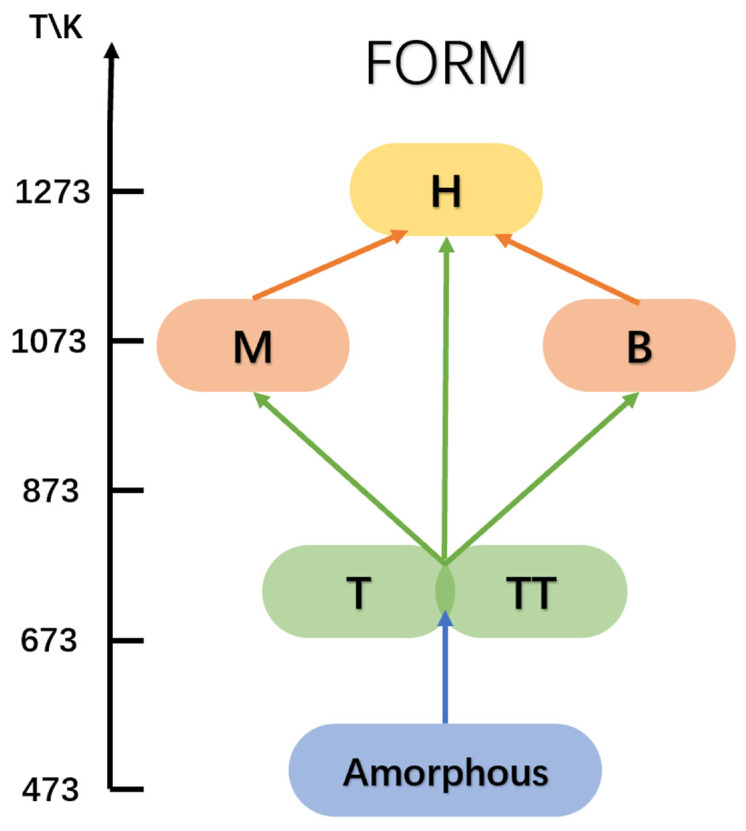
Nb_2_O_5_ crystal phase with temperature.

Amorphous Nb_2_O_5_ is typically obtained through various low-temperature synthesis methods and can be subsequently crystallized into either the TT or T phase at approximately 500 °C (Figure 2). At intermediate temperatures (~800 °C), the material can transform into the M phase (tetragonal), while the H phase forms at ≥1000 °C. It is important to note that, besides temperature, other factors can also influence the formation of Nb_2_O_5_ crystals. The phase stability is affected by environmental temperature and pressure, while the existence of polymorphs depends on the heating and preparation methods [22]. However, the nature of the starting material and the presence of impurities are also important factors affecting the formation of Nb_2_O_5_ crystals [11,23]. As a result, temperature should be considered an indicative factor rather than the sole determinant of the crystalline phase of Nb_2_O_5_.

The different polymorphs of Nb_2_O_5_ include distorted octahedra (NbO_6_); the degree of distortion is influenced by the type of connection between the octahedra, whether through edges, angles or a combination of both [24]. Schäfer et al. [11] identified multiple modes of attachment between the octahedra while maintaining an O/Nb ratio of 2.5, confirming the existence of various arrangements of Nb_2_O_5_ and polycrystalline forms of niobium oxide. Nb atoms exhibit 6-fold (NbO_6_) and 7-fold (NbO_7_) coordination in the T and TT phases, along with distorted octahedral and pentagonal bipyramidal sites.

Undoubtedly, different crystalline phases of Nb_2_O_5_ exhibit different properties. For instance, the dielectric constant of the H phase can reach 100. The T-phase has applications in electrochemistry owing to its excellent electrochemical properties, including its resistance to reactions with other substances and cyclic stability. Extensive research has also been conducted to assess the application of the TT phase as an electrochromic material.

#### 2.1.1. T- Nb_2_O_5_

**Figure 3:**
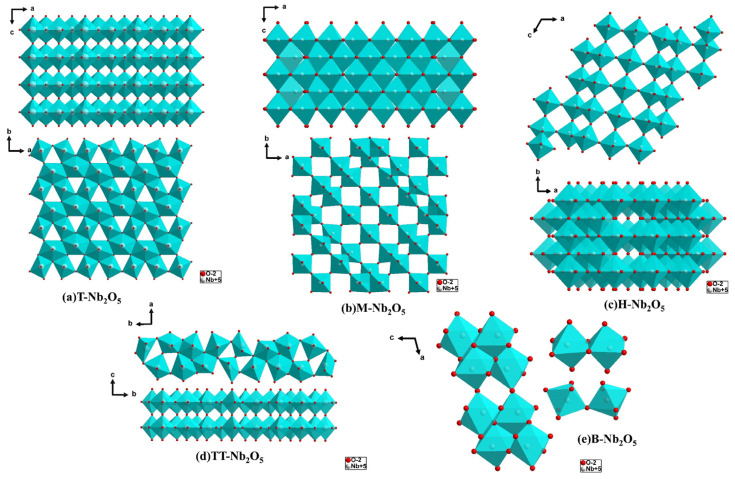
Crystal structures of Nb_2_O_5_ materials. (**a**) Structure of T-Nb_2_O_5_ observed along the b-axis and c-axis. (**b**) Structure of M-Nb_2_O_5_ observed along the b-axis and c-axis. (**c**) Structure of H-Nb_2_O_5_ observed along the b an c axes. (**d**) Crystal structure of TT-Nb_2_O_5_ in different directions. (**e**) Unit cell of B-Nb_2_O_5_ phase, On the right side of the picture it is shown how the polyhedra are connected in the crystal structure.

The T-Nb_2_O_5_ phase possesses an orthorhombic crystal structure with a Pbam space group. In its conventional cell, it comprises 16.8 Nb and 42 O atoms. Among these, sixteen Nb ions occupy four Wyckoff positions 8i with half occupancy, while eleven O atoms are distributed across one 2b, four 4g, and six 4h positions. The remaining 0.8 Nb atoms exhibit random occupancies of 0.08, 0.08, and 0.04 across three 4g Wyckoff positions. The orthorhombic T-Nb_2_O_5_ phase comprises Nb atoms surrounded by six or seven oxygen atoms, forming twisted octahedral or pentagonal bipyramidal shapes [25] (Figure 3a). In the T and TT phases, Nb atoms have six-fold (NbO_6_) and seven-fold (NbO_7_) coordination, which can lead to the formation of distorted octahedrons and pentagonal bipyramids, respectively [24] (Figure 3a,d).

Serghiou et al. first discovered that the T phase of Nb_2_O_5_ becomes amorphous under certain conditions, including pressure and temperatures up to 19.2 GPa and 300 K, respectively. During this amorphous process, the oxide becomes amorphous and reduced [26].

In the 2010s, T-Nb_2_O_5_ gradually became a research hotspot. Li et al. reported a chemical method without catalyst topology, combined with molten salt synthesis (MSS), to achieve the large-scale synthesis of rod-like H-Nb_2_O_5_ and sheet-like T-Nb_2_O_5_ single crystals. Subsequently, Raman spectroscopy, SEM, X-ray diffraction, and TEM were employed to investigate the structural changes involved in the process [27].

In the mid-2010s, research on applying T-Nb_2_O_5_ intercalated pseudocapacitors gained popularity. Augustyn et al. recently demonstrated that mesoporous and nanocrystalline films of T-Nb_2_O_5_ (i.e., trapezoidal Nb_2_O_5_) exhibit behavior consistent with pseudocapacitance upon inserting lithium ions [28]. Meanwhile, Kong et al. employed a hydrothermal synthesis technique to anchor T-Nb_2_O_5_ nanocrystals onto conductive graphene sheets, fabricating asymmetric SCs. The T-Nb_2_O_5_/graphene nanocomposites and mesoporous carbon were used as negative/positive electrodes and exhibited high-rate responses, significantly enhancing the performance of SCs [29]. According to Lim et al., The Nb_2_O_5_@C NCs core-shell nanocrystals exhibit excellent electrochemical performance. More specifically, the core-shell structure provides a high surface area and efficient electron/ion transport pathways, with the carbon shell acting as a buffer to accommodate volume changes during cycling. The one-pot synthesis method offers a simple and scalable approach for producing high-performance anode materials for next-generation LIBs [30].

In the late 2010s, T-Nb_2_O_5_ gained attention as a promising anode material for sodium-ion batteries (NIBs) due to its unique surface frame and large interplanar lattice spacing [31]. In addition, research into applying T-Nb_2_O_5_ in potassium-ion batteries (KIBs) began. For the first time, Li et al. studied nanostructured T-Nb_2_O_5_ as a cathode material for KIBs [32].

During the late 2010s and early 2020s, most studies focused on modifying the T-Nb_2_O_5_ anode and cathode of hybrid supercapacitor (HSC) or optimizing synthesis methods to improve its defects. Additionally, the application of T-Nb_2_O_5_ in lithium-sulfur batteries was explored. Wang et al. [33] reported on a novel niobium oxide matrix that incorporates amorphous/crystalline hetero-conjunctions (A/T-Nb_2_O_5_), serving as a two-in-one host for a Li-S system. This matrix significantly improves the electrochemical performance of the Li-S system, with A-Nb_2_O_5_ providing a high surface area for sulfur adsorption and T-Nb_2_O_5_ offering a stable framework for sulfur confinement. The amorphous/crystalline hetero-conjunctions further enhance the conductivity of the matrix and facilitate the transport of lithium ions, presenting a promising strategy for developing high-performance electrode materials in next-generation energy storage systems.

#### 2.1.2. TT-Nb_2_O_5_

**Figure 4:**
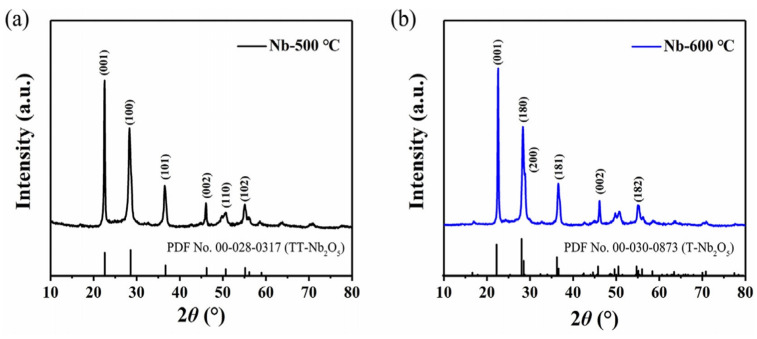
XRD patterns of various structures of Nb_2_O_5_ materials. Different niobium oxide phases (**a**) TT-Nb_2_O_5_ and (**b**) T-Nb_2_O_5_ were obtained by oxidizing niobium powder at 500 and 600 °C, respectively. Reprinted with permission from Ref. [34], Copyright 2020 Elsevier.

At low temperatures, the two phases of Nb_2_O_5_, TT, and T, share many similarities. For example, the X-ray diffraction patterns of the TT and T phases of Nb_2_O_5_ exhibit remarkable resemblance (Figure 4a,b). However, a key distinguishing factor is that the XRD pattern of the TT-Nb_2_O_5_ phase includes a peak corresponding to a split reflection, which is not present in the T-Nb_2_O_5_ phase [1,23]. The emergence of the TT phase of Nb_2_O_5_ is not necessarily associated with using pure components as starting materials. Rather, TT may represent a less crystalline form of T, possibly stabilized by impurities [23]. In certain cases, oxygen atoms within Nb_2_O_5_ can be substituted by monovalent species like OH^−^ or Cl^−^ or even vacancies. This substitution process helps stabilize the TT phase of Nb_2_O_5_.

In TT-Nb_2_O_5_, the unit cell of Nb_2_O_5_ contains half of its formula equivalent, along with a constitutional defect involving the absence of one oxygen atom per unit cell [25] (Figure 3d). The Nb atom is positioned at the center of 4, 5, or 6 oxygen atoms within the ab plane. The crystal structure of Nb_2_O_5_ along the a-axis comprises two in-plane niobium layers interconnected by oxygen bonds, forming a relatively stacked structure. Meanwhile, the b-axis exhibits a tunnel-like structure with an approximate diameter of 4 Å, extending throughout the entire structure. This unique feature of Nb_2_O_5_ is responsible for its enhanced ion diffusion properties [35,36,37]. Along the c-axis, the crystal structure of Nb_2_O_5_ exhibits a disordered hexagon formed by oxygen atoms in six faces and an Nb-O-Nb-O chain structure. The absence of oxygen in the structure leads to the deformation of these polyhedra.

During the 1950s, Frevel and Rinn conducted studies on Nb_2_O_5_ and discovered a “low temperature” phase, now known as TT-Nb_2_O_5_. Their research utilized X-ray powder diffraction and identified a pseudo-hexagonal phase with lattice constants a = 3.607 Å and c = 3.925 Å. This phase transforms into T-Nb_2_O_5_ when heated to 973 K. Since its discovery, TT-Nb_2_O_5_ has been the subject of considerable scientific debate. Some believe the TT phase is merely a variant of T-Nb_2_O_5_, suggesting it is less crystalline than T-Nb_2_O_5_ [38]. Others say it is not even a separate phase [39]. Meanwhile, Terao was the first to identify the orthogonal structure of T-Nb_2_O_5_ and suggested that TT-Nb_2_O_5_ is a metal oxide with crystallization defects instead of a poorly crystallized T-phase [40]. Tamura suggested that TT-Nb_2_O_5_ possesses monoclinic cells and is stabilized by trace impurities in the sample [41]. Weissman et al. proposed that the structures of TT-Nb_2_O_5_ and T-Nb_2_O_5_ are highly similar, noting the absence of peak splitting and the presence of broad peaks in TT-Nb_2_O_5_, indicating that niobium resides in a more symmetrical 4h Wyckoff position rather than the 8i position found in T-Nb_2_O_5_ [42]. Their model suggests that TT-Nb_2_O_5_ can be indexed within the orthorhombic system of T-Nb_2_O_5_ and comprises subcell domains within a superlattice. Despite the distortions leading to unusual crystallization, TT-Nb_2_O_5_ displays pseudo-hexagonal subcell symmetry, while the superlattice follows an orthonormal crystal system [42]. Recently, Košutová et al. demonstrated an improved TT structure using hexagonal cells [43]. TT-Nb_2_O_5_ exhibits a complex structure with a disordered pseudohexagonal subcell of β = 120°, resulting in an orthogonal superlattice that resembles the T-phase. Niobium atoms occupy a more symmetrical Wyckoff position in this phase. The disordered polyhedron in TT-Nb_2_O_5_ forms a tunnel structure along the b-axis. Given its significance in the performance and applications of TT-Nb_2_O_5_, further investigation into its structure is warranted [44].

#### 2.1.3. H-Nb_2_O_5_

H-Nb_2_O_5_ exhibits a high degree of order, with its structure divided into blocks [23]. In this monoclinic lattice, H-Nb_2_O_5_ comprises groups of ReO_3_-type blocks, encompassing 3 × 4 and 3 × 5 blocks. These blocks are arranged in a layered structure, with the 3 × 4 blocks forming the first layer and the 3 × 5 blocks forming the second layer. It contains the NbO_6_ octahedron [25] (Figure 3c). The ReO_3_-type blocks in H-Nb_2_O_5_ are coupled by edge-sharing and move half-unit cell size along the c-axis. The NbO_6_ units are connected through shared angles within a block [25]. For every 28 Nb atoms in the cell, one resides at the tetrahedral site, typically at certain block junctions.

This variant of Nb_2_O_5_ is readily available. Indeed, H-Nb_2_O_5_ can be produced in any other form when heated in air to ~1100 °C. If M-type single crystals are heated at 1100 °C, they retain their single-crystal properties during the transition to H-type, whereas all other Nb_2_O_5_ crystals convert to polycrystalline H-Nb_2_O_5_ [11].

H-Nb_2_O_5_ was initially identified as a high-temperature variant of Nb_2_O_5_ by Brauer in 1941. Later, in 1964, Gatehouse and Wadsley determined the crystal structure of H-Nb_2_O_5_ using Patterson and differential synthesis methods. Subsequently, Busing et al. used ORFLS to refine the structure by the full matrix least square method. Kato conducted additional refinements of the H-Nb_2_O_5_ structure in 1975 [16]. In the 21st century, extensive research has been conducted on the applications of H-Nb_2_O_5_.

#### 2.1.4. M-Nb_2_O_5_

M-Nb_2_O_5_ shares a crystal structure similar to H-Nb_2_O_5_ and contains 4 × 4 ReO_3_-type blocks. The close resemblance between M- and H-type X-ray powder patterns accounts for this similarity.

M-Nb_2_O_5_ belongs to the tetragonal system with lattice constants a = 20.44 Å and b = 3.832 Å. The space group is I4/mmm. Presumed metastable variants of Nb_2_O_5_ can be obtained through various pathways within the temperature range of ~900 °C. Single crystals are obtained using chemical transport techniques. In the structure of M-Nb_2_O_5_ (Figure 3b), Nb-O octahedrons are arranged in a blocky manner and can be considered part of the ReO_3_ lattice. Formally, the structure of M-Nb_2_O_5_ is derived from that of N-Nb_2_O_5_. Perpendicular to the C-axis, the dimension of the block measures 4 × 4 octahedral diagonal units. In this plane, adjacent blocks are connected by edges. In the c direction, the octahedrons are connected by angles. In M-Nb_2_O_5_, four structures are interconnected to form a set of four edge-connected octahedrons (NbO_4/4_O_2/2_).

#### 2.1.5. B-Nb_2_O_5_

B-Nb_2_O_5_ features a monoclinic crystal structure indexed in the space group C2/c, with four formula units in each cell. The structure comprises eight cations (Nb) located at the 8f Wyckoff position and 20 anionic oxygen ions (O) located at three Wyckoff locations: 4e (O_1_) and two 8f (O_2_ and O_3_). The crystal structure of the B-Nb_2_O_5_ phase consists of twisted NbO_6_ octahedral blocks arranged in a string of pairs of shared edge octahedra connected in a zigzag pattern of shared angle octahedrons (Figure 3e). According to the study of Pinto et al., DFT calculations and experimental evidence confirm that the B phase is the more stable phase at lower temperatures [14].

#### 2.1.6. Other Nb_2_O_5_ Phases

In addition to the common crystal phases of Nb_2_O_5_, there are several less common or less studied phases, including N-Nb_2_O_5_, P-Nb_2_O_5_, and R-Nb_2_O_5_, among others. The presence of twin domains observed in previous studies likely results from the crystal structure and growth conditions during synthesis. The presence of coherent twins at 90° from each other suggests that the crystal structure of N-Nb_2_O_5_ possesses a high degree of symmetry. Regions containing blocks of different sizes may be attributed to defects or impurities within the crystal structure. Moreover, N-Nb_2_O_5_ and M-Nb_2_O_5_ differ only in structural arrangement. The synthesis of N-Nb_2_O_5_ was first investigated by Schafer et al. in 1964 [45], with its crystal structure reported by Andersson et al. in 1967 [17]. Meanwhile, Uyeda et al. (1984) [46] had earlier published HRTEM images of N-Nb_2_O_5_ and utilized the compound as a standard specimen; however, they did not describe the various types of twins in N-Nb_2_O_5_. Overall, these studies have provided insights into the microstructure of N-Nb_2_O_5_, emphasizing the importance of understanding crystal structure and defects in materials science.

RS-Nb_2_O_5_ is a nanostructured rock salt Nb_2_O_5_ electrode, reported by Barnes et al. [47]. This crystalline phase has excellent electrical properties through the amorphous to crystalline transition during the repeated electrochemical cycle with Li^+^. 

### 2.2. Synthesis Methods

Researchers have taken a keen interest in Nb_2_O_5_, a non-toxic substance with ecological friendliness and a robust oxidation capacity. Due to its broad potential applications, extensive investigations have been performed to explore the various preparation methods for Nb_2_O_5_. The primary motivation for selecting the appropriate synthesis method lies in its capability to modify the properties of Nb_2_O_5_, particularly its crystal phase and morphology.

**Figure 5:**
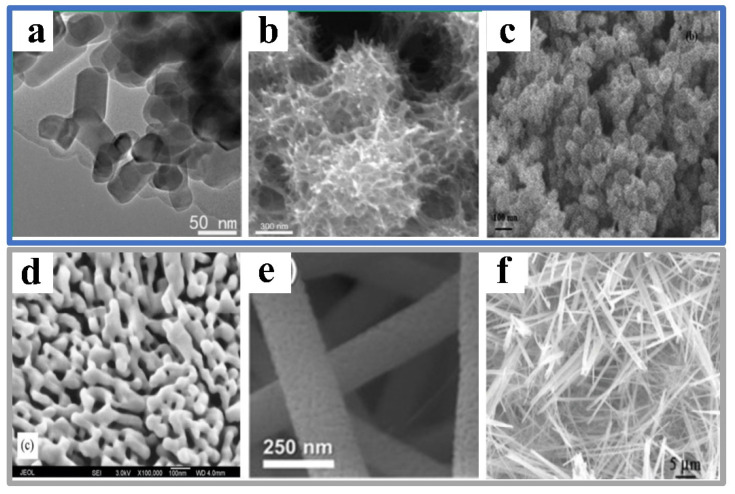
(**a**) TEM images of Nb_2_O_5_ nanorods synthesized by the hydrothermal method. Reprinted with permission from Ref. [48], Copyright 2017 Elsevier. Hydrothermal method (**b**) Nb_2_O_5_ nanostructure. Reprinted with permission from Ref. [49], Copyright 2018 American Chemical Society. Precipitation method (**c**) porous Nb_2_O_5_ nanoparticles. Reprinted with permission from Ref. [50], Copyright 2007 Elsevier. (**d**) N2-650 samples prepared by the sol-gel method. Reprinted with permission from Ref. [51], Copyright 2004 Elsevier. (**e**) High power SEM image of m-T-Nb_2_O_5_ NFs prepared by electrospinning. Reprinted with permission from Ref. [52], Copyright 2017 Elsevier. (**f**) Electrostatic spun multiple NaNbO_3_/Nb_2_O_5_ heterostructure nanotubes. Reprinted with permission from Ref. [53], Copyright 2010 WILEY-VCH Verlag GmbH & Co. KGaA, Weinheim.

Significant attention has been devoted to developing niobium oxide films or particles, revealing their unique properties. Various techniques have been employed, including pulsed laser decomposition (PLD), electrodeposition, magnetron sputtering, plasma immersion ion implantation, and the sol-gel process. These methods have been utilized to prepare Nb_2_O_5_ nanostructures. Indeed, the characteristics of the surface nanostructures formed by these different synthesis methods are unique (Figure 5). For example, the hydrothermal method can be used to synthesize Nb_2_O_5_ nanorods and sea urchin nanostructures (Figure 5a,b). Meanwhile, the precipitation rule formed a nanosphere layer with a porous structure (Figure 5c). The samples prepared by the sol-gel method are coral-like nanostructures (Figure 5d), whereas longer Nb_2_O_5_ nanowires are synthesized by electrospinning (Figure 5e). Still further, filamentous nanostructures with chaotic surfaces can be formed via electrospinning (Figure 5f). Additionally, a self-organized microstructure of niobium oxide has been produced through potentiostatic anodization.

#### 2.2.1. Hydrothermal and Solvothermal Methods

The hydrothermal and solvothermal methods are widely used due to their simplicity, low cost, and high yield. Typically, the reaction occurs within an autoclave or a Teflon-lined stainless steel vessel filled with water or organic solvents. The Nb ion arises from the reaction of niobium with acidic or basic solutions or by dissolving niobium salts; the solution is then heated to 100–600 °C for several hours or even days, during which Nb_2_O_5_ nanostructures grow [54,55,56]. Nb_2_O_5_ polycrystal hollow nanospheres and single-crystal nanotubes have been prepared by adjusting the molar ratio of Nb/Ti and the amount of F^−^ ions used [56]. Meanwhile, tree-like Nb_2_O_5_ nanotrees have been fabricated by secondary nucleation of Nb_2_O_5_ nanowires under hydrothermal conditions [57]. Additionally, monoclinic Nb_2_O_5_ nanotube arrays from pseudo-hexagonal Nb_2_O_5_ nanorod arrays have been reported. The phase transformation was due to energy differences between the pseudo-hexagonal and monoclinic Nb_2_O_5_ nanostructures [58]. Future developments may further optimize preparation methods and properties of Nb_2_O_5_ nanoparticles, potentially through doping with other elements or surface modifications with organic molecules to improve their electrochemical properties.

#### 2.2.2. Anodization Method

The anodization method is a prevalent nanofabrication technique renowned for creating highly porous and well-ordered oxide structures. The anodization process is initiated by immersing the working and counter electrodes (usually a platinum sheet) in an electrolyte. Current and voltage are then applied, initiating chemical reactions at the interface between the electrolyte and electrode, forming a thin film structure. The shape and size of the resulting film are related to myriad factors, including the electrolyte composition, electrolyte temperature, amount of current or voltage applied, and the conduction time. 

Anodized films are typically amorphous, however, they can be crystallized through annealing. Research on anodized Nb_2_O_5_ film dates back to the 1960s. Draper and colleagues studied the formation of oxide films on niobium tablets, investigating how the electrolyte’s composition affects the film material’s growth, particularly with regard to structural irregularities [57]. Subsequently, studies reported on the properties of anode Nb_2_O_5_ films (i.e., resistivity, dielectric constant). Other initial investigations into the surface morphology of anodized Nb_2_O_5_ and crystal analysis using electron microscopy electron beam crystallization have also been documented [58,59].

The anodization process of Nb has been extensively investigated using various electrolytes, including sulfuric acid, phosphoric acid, NaOH, Na_2_CO_3_, HF, glycerol, and phosphate-based solutions. These studies aimed to understand the electrochemical behavior and optimize the formation of oxide layers on the Nb surfaces for different applications. Most reported anodic Nb_2_O_5_ films possess a highly nanoporous structure on an Nb foil substrate [60]. One of the most significant studies in this area was conducted by Habazaki and colleagues, who discussed the influence of water content and elevated temperatures on the formation of porous Nb_2_O_5_ during the anodization process using a K_2_HPO_4_-glycerol electrolyte. They explored how these factors affect the morphology, structure, and properties of the resulting porous Nb_2_O_5_ films [60]. They reported that maintaining the water content of the electrolyte at 0.08 mass% at 160 °C significantly increased the film’s thickness. This was attributed to intensified field strength arising from the heightened concentration of phosphorus species in the electrolyte solution. Meanwhile, Ou and colleagues developed an Nb_2_O_5_ crisscross nanoporous network by high-temperature anodization [61]. 

Throughout these processes, ion diffusion during anodization is affected by several factors. Apart from nanoporous structures, microcones can also be produced through Nb anodization in an electrolyte solution with low hydrofluoric acid content in deionized water or a glycerol electrolyte solution containing K_2_HPO_4_ [62,63]. Wei and colleagues improved the anodic oxidation of Nb in NH_4_F-based glycerol electrolytes and successfully fabricated Nb_2_O_5_ nanotubes up to 4 mm [64]. Lee et al. first reported the anodic oxidation process of highly ordered Nb_2_O_5_ nanochannels grown at 180 °C in a glycerol electrolyte solution containing K_2_HPO_4_ [65]. Lee et al. preheated the electrolyte to 200 °C before anodic oxidation to reduce the water content and successfully grew Nb_2_O_5_ thick nanochannel films. Similarly, Abdul Rani and colleagues reported the synthesis of Nb_2_O_5_ thin films on anodic nanochannels [66].

Other studies have reported on the production of nanoporous and nanochannel Nb_2_O_5_ thin films through Nb thin film anodization and deposition via RF sputtering on FTO glass substrates [67,68]. For the first time, Liu and colleagues successfully demonstrated the preparation of Nb_2_O_5_ nanotube powders by a simple electrochemical anodization method. In the anodizing process, the anodic oxide was released continuously and spontaneously into the electrolyte, which was collected to produce a white powder. The resulting powder primarily comprised nanotubes, with lengths ranging from ~50 to 100 nm and diameters of approximately 20 to 30 nm [69]. Meanwhile, Khairir et al. prepared a nanoporous thin film structure of Nb_2_O_5_ by the anodic oxidation method under different oxidation times; longer anodic oxidation times led to larger pore sizes [70].

**Figure 6:**
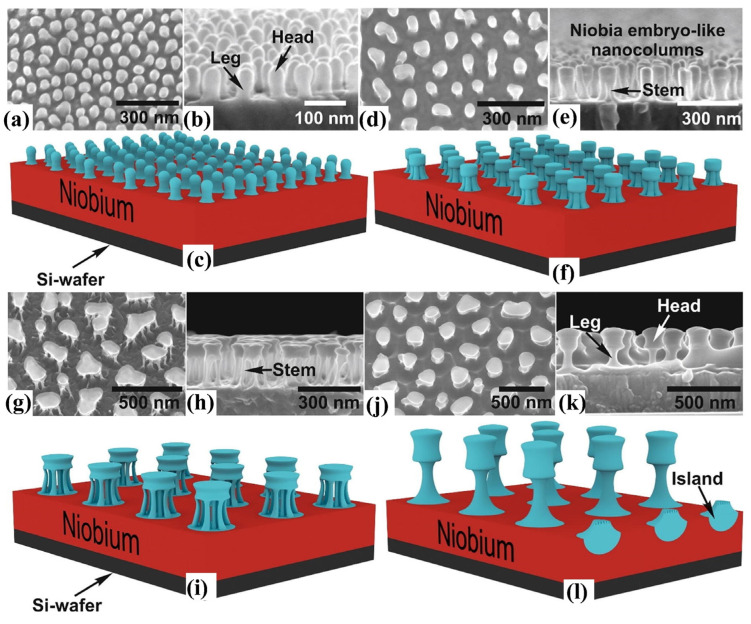
SEM views of surface and cross-sectional, schematic 3D views (**a**–**c**) skittle- and (**d**–**f**) medusa-like embryos formed by anodizing bilayer Al/Nb systems in 0.4 mol dm^−3^ oxalic acid aqueous solution at 37 and 53 V. SEM views of surface and cross-sectional, schematic 3D views (**g**–**i**) medusa—and (**j**–**l**) goblet-like embryos formed by anodizing bilayer Al/Nb systems in 0.4 mol dm^−3^ phosphoric acid aqueous solution at 100 and 150 V. The images were obtained after the alumina layer had been dissolved away (“alumina-free” samples). Reprinted with permission from Ref. [73], Copyright 2021 Elsevier.

Pligovka et al. conducted X-ray diffraction analysis on niobium oxide nanostructures, including defective non-uniform arrays and nanocolumns, fabricated via electrochemical anodization [71]. Gorokh et al. further studied the composition of columnar niobium oxide nanostructures (CNONS), synthesized initial samples by vacuum magnetron sputtering Nb/Al targets on a silicon substrate, and then synthesized samples by anodization and repeated anodization (reanodization) in different electrolytes. Finally, the growth mechanism of CNONS was proposed by analyzing the samples. The infrared spectroscopy analysis of CNONS identified three oxide phases in the NB-O system: NbO, NbO_2,_ and Nb_2_O_5_. Moreover, the top of the column was composed primarily of two Nb_2_O_5_ modifications: α-Nb_2_O_5_ and β-Nb_2_O_5_ [72]. In 2021, Pligovka et al. analyzed the morphology of niobium oxide nanocrystals formed by an electrochemical anodizing Al/Nb system with a similar synthesis method. They formed three different embryonic morphological types: skittle-, medusa- and goblet-like (Figure 6). Moreover, the morphological characteristics of the region between the top and bottom of the formed nanostructures were closely related to the electrolyte and the formation voltage of the anodic oxidation process [73].

The electrophysical properties of columnar niobium oxide nanostructures have also been studied. The measurement results showed that the current-voltage I-U curve is nonlinear and asymmetrical. An increase in temperature resulted in an increase in current. This behavior may represent a *p-n* junction or a metal-semiconductor junction [74]. Recently, its application in nano-optical biosensors has also been preliminarily studied [75].

#### 2.2.3. Sol-Gel Methods

Since the sol-gel method was discovered in the 1970s, it has been rapidly developed and successfully applied in various material engineering fields [76]. Ulrich in 1988 [77] and Hench in 1990 [78] described the broad prospect of this method. To date, its application has been extensive, particularly in wet chemistry, as it allows for the precise control of composition and homogeneity during material synthesis at low temperatures. Furthermore, it offers a simple process with the potential for cost-effective large-scale production.

To generate sols, alcohol solutions are typically hydrolyzed with water, followed by polymerization or aggregation to produce dispersed fine particles. Depending on the desired sol composition and properties, these precursor compounds can be inorganic or metal-organic. The fundamental chemistry of sol-gel processes has been thoroughly covered in other reviews [79]. As per the summary of Bokov et al., during the sol-gel process, molecular precursors such as metallic alkoxides undergo hydrolysis or alcoholysis, resulting in gel formation from solutions in water or alcohol via heating and agitation. Since the gels resulting from these processes usually retain some water and solvent, it is necessary to dry them using appropriate methods that align with their intended properties and applications. For example, if an alcohol solution is utilized, drying can be accomplished by burning off the alcohol. Once dried, the resulting gel is often powdered and subjected to calcination [80]. In brief, the sol particles connect, forming a network of inorganic polymers known as gels, which retain some residual water and solvent. A dry gel is formed or coated in a transition window from sol to gel before removing the remaining water and solvent. The gel is then heat-treated to form the final dense product. Depositing oxide films using the sol-gel method is typically accomplished through dipping or spin coating techniques. However, the sol-gel method does have certain limitations, including the potential for uneven film thickness due to weak bonding. Moreover, it is difficult to control the reaction rate and porosity [81].

Alquier et al. first reported the preparation of Nb_2_O_5_ salt and its solvent by the sol-gel method in 1986 [82]. Post-annealing treatment is typically necessary to induce the crystallization of Nb_2_O_5_ films prepared via these processes. Schmitt et al. prepared these thin films by dissolving NbCl_5_ powder in a mixture of butanol and acetic acid [83]. Following the preparation of the Nb_2_O_5_ film by dip-coating, the Nb_2_O_5_ coating was annealed at different temperatures from 400 to 600 °C, causing its structure to shift from amorphous to crystalline (i.e., a TT structure). Subsequently, others have demonstrated analogous synthetic procedures using alternative precursors, such as niobium ethanol and ammonium niobium oxalate. For instance, Melo et al. prepared Nb_2_O_5_ and Nb_2_O_5_: Li^+^ films by the sol-gel method with acoustic catalysis. They employed NbCl_5_ as the precursor and butanol as the solvent, adding lithium salt LiCF_3_SO_3_ into the precursor solution to obtain thin films with different electrochemical properties [84]. In 2021, Xu et al. studied the impact of crystallinity on the optical properties of sol-gel-prepared Nb_2_O_5_. TG-DSC analysis revealed amorphous Nb_2_O_5_ up to 460 °C. Meanwhile, XRD results indicated the transformation of amorphous Nb_2_O_5_ into a pseudo-hexagonal phase, with higher temperatures resulting in improved crystallinity. UV-VIS and Raman spectra results demonstrated a progression in the arrangement of Nb_2_O_5_ atoms, starting with short-range order and progressing to medium-range, ultimately reaching a long-range order. That is, as the temperature increased, atoms became long-range ordered structures by connecting structural units [85].

#### 2.2.4. Electrodeposition

Electrodeposition is widely used to manufacture nanostructured materials in various forms, such as powder, composite, and thin films. It is a well-established technique that produces a thin and uniform coating by applying an electric potential between two electrodes immersed in a solution, causing metal ions to deposit onto a substrate. In contrast to the anodization method described in Section 2.2.2, the metal oxide film formed by electrodeposition is at the cathode. Electroreduction is commonly used to produce low-cost (metal) materials, while electrooxidation is typically employed for high-value material production [86].

The electrodeposition process can be tailored by adjusting several parameters, including current density/voltage, temperature, and the addition of agents to the electrolyte solution. Additionally, modifying the substrate’s surface properties can achieve the desired electrodeposition characteristics. The current density/voltage pair is pivotal in controlling electrodeposition characteristics and determining the threshold for different reactions. A significant benefit of electrodeposition is the ability to regulate the reaction using an external circuit, enabling accurate and simple control over material deposition [86]. Niobium ions and hydrogen peroxide are typically required in the aqueous electrolytes for the electrodeposition of Nb_2_O_5_ [87]. 

Cathodic electrodeposition of niobium oxide (NbO_x_) is hindered by Nb^3+^’s extremely negative reduction potential (−1.1 V vs. NHE), leading to the coevolution of destructive H_2_ gas when the reaction occurs in aqueous solutions [88]. Crayston et al. [89] avoided this issue by using electrically generated OH^−^ to electrodeposit NbO_x_, precipitating niobium ions, and immobilizing them in porous films prepared using the sol-gel technique. Meanwhile, in the late 1990s, Zhitomirsky electrodeposited Nb_2_O_5_ thin films on platinum-coated silicon or platinum substrates using peroxide as a precursor. They applied a stable 20 mA cm^−2^ current during the 20-min electrodeposition process, which occurred at 1 °C. The electrolyte mixture comprised NbCl_5_ and H_2_O_2_ [90,91]. The prepared Nb_2_O_5_ film had a uniform thickness and strong adhesion to the underlying material surface. Electrodeposition can also be performed using non-aqueous electrolytes, although a supporting electrolyte is typically required to facilitate current conduction in these systems.

In the study by Kamada et al., Nb served as the anode and platinum as the cathode. Electrodeposition was carried out in 0.01 M I^2−^ or Br^2−^ acetone as the solvent under a constant pressure of 50 V at room temperature and direct current. Introducing iodine to acetone led to anodization of the metal anode, however, electrochemical dissolution of the anode and its cathodic deposition did not occur. Conversely, including bromine in the solution promoted anodic dissolution and electrodeposition [92]. Meanwhile, Zhao et al. [93] applied the electrodeposition method to deposit T-Nb_2_O_5_ quantum dots onto a Ti nanorod array, creating a Ti@T-Nb_2_O_5_ core-shell array electrode with good electrical properties. The size of T-Nb_2_O_5_ particles can be manipulated by modifying the deposition current density. For example, with a deposition current of 6 mA cm^−2^, quantum dots measuring several nanometers in diameter can be synthesized. Jha et al. improved Zhitomirsky’s electrodeposition method to obtain NbO_x_ colloids by rapidly injecting methanol-dissolved niobium salt into a hydrogen peroxide solution cooled to approximately 2 °C. This led to the production of a pure phase T-Nb_2_O_5_ film with a uniform and controllable thickness, high porosity, and good cycling efficiency [88].

#### 2.2.5. Vapor Phase Deposition

Vapor deposition is a technique used to produce material layers by condensing vaporized source material under specific conditions. Vapor deposition can be classified into two primary categories: physical vapor deposition (PVD) and chemical vapor deposition (CVD). PVD involves evaporating solid or liquid material sources in a vacuum environment. This results in the formation of gaseous atoms, molecules, or partially ionized ions. These particles are then deposited onto the substrate surface as a gas (in a low-pressure state) or as plasma, producing films with different functions [94,95]. The PVD methods include vacuum evaporation, sputter coating [96], ion beam-assisted deposition [97,98], thermal evaporation [99], pulsed laser deposition (PLD) [100], etc. PVD technology has advanced to the point where it is possible to deposit various films, including those composed of metals, alloys, compounds, ceramics, semiconductors, and polymers, among other materials.

Sputtering is employed to prepare Nb_2_O_5_ thin films as it can use a direct current (DC) [95,101] or radio frequency (RF) [102,103,104,105] power supply in an oxygen (O_2_) environment with metal niobium (Nb) [106] or Nb_2_O_5_ [102,103,105] as the target, using argon (Ar) and other carrier gas preparations. By manipulating the deposition parameters, such as pressure, target-substrate distance, substrate temperature, discharge voltage, and RF power, these methods can produce Nb_2_O_5_ thin films with precisely controlled size, crystallinity, and grain size [107]. Meanwhile, post-annealing treatment can improve the crystallinity of sputtered films. However, the deposition rate of this technique is low and time-consuming. However, further investigations have revealed that using pulsed magnetron sputtering and PLD technology, Nb_2_O_5_ thin films can be deposited at tens of microns per hour while maintaining the original stoichiometric ratio of the bulk target material.

CVD is a widely-used technique involving chemical reactions of gaseous or vapor-state substances to form solid deposits at the interface between gas and solid or between two gas phases. The main difference between CVD and PVD is that chemical reactions occur during CVD deposition. CVD is a gas-phase chemical growth process wherein various raw gas materials are introduced into a reaction chamber. Chemical reactions occur among the gases, generating new materials, subsequently deposited onto the substrate surface. As such, CVD is an effective method for preparing Nb_2_O_5_, allowing precise control over the thickness, morphology, shape, and composition of Nb_2_O_5_ films by regulating reaction conditions to meet diverse application requirements. In addition, CVD technology is widely applied in fabricating thin films, particularly conformal coatings and nanostructures [108]. Various CVD techniques include atmospheric pressure chemical vapor deposition (APCVD), low-pressure chemical vapor deposition (LPCVD) [109], vapor phase epitaxy (VPE) [110], ultra-high vacuum chemical vapor deposition (UHVCVD) [111], laser-induced chemical vapor deposition (LCVD) [112], among others.

Typically, Nb_2_O_5_ films are deposited using CVD by introducing precursor chemicals like NbCl_5_ and pentaethoxy niobium [Nb(OC_2_H_5_)_5_] into a reaction chamber via a carrier gas. They undergo thermal decomposition on the surface of a heated substrate to form the desired film. O’Neill et al. reported that the growth of Nb_2_O_5_ films via CVD is significantly influenced by the substrate’s temperature [113]. Other studies using the CVD method provide an excellent alternative to the production of layered Nb_2_O_5_, especially for fiber coating applications [114]. For example, Silveira et al. exposed glass fiber to air after reacting with NbCl_5_, heat treating at 300 °C for 2 h, and heat treating and hydrolyzing in water to achieve a well-coated glass fiber with Nb_2_O_5_ [115].

Spray pyrolysis is another technique commonly employed to produce Nb_2_O_5_ films with various thicknesses through an aerosol-assisted CVD process [116,117]. This method features simple experimental equipment and high production efficiency. However, when depositing Nb_2_O_5_ films at low temperatures, post-annealing treatment is often required to induce crystallization and improve the structural quality of the films.

It is worth mentioning that HV-CVD is also an effective method for synthesizing Nb_2_O_5_ thin films [108]. According to Yury Kuzminykh’s research, HV-CVD technology offers unique advantages in Nb_2_O_5_ preparation, achieving deposition rates up to 500 nm/h, markedly higher than that of the MBE process. The film thickness of vegetation by this method is up to several microns. Moreover, HV-CVD requires lower substrate temperatures than MO-CVD, opening the possibility for in-situ high vacuum characterization techniques [108].

#### 2.2.6. Thermal Oxidation

The preparation of Nb_2_O_5_ via the thermal oxidation process is typically a straightforward procedure involving using Nb metal/foil or powder as the starting material. The material is subsequently loaded into a furnace and heated in a high-oxygen or pure O_2_ environment to temperatures up to 1000 °C to facilitate oxidation and the formation of Nb_2_O_5_ [118,119,120]. The thermal oxidation process relies on the diffusion of oxidizing agents like O_2_ onto the substrate at elevated temperatures. These agents then react with the Nb material, leading to the direct creation of nanoscale Nb_2_O_5_ structures on the surface. This approach enables the fabrication of Nb_2_O_5_ nanowires, which can extend to lengths up to 20 mm [121]. The resulting nanostructures are influenced by factors such as temperature, time, metal catalysts and gas atmosphere.

## 3. Properties of Nb_2_O_5_

In the following section, other basic properties of Nb_2_O_5_ including electrical, optical and thermal properties will be discussed.

### 3.1. Electrical Properties

The properties of metal oxides play a critical role in developing electronic devices, with key parameters such as band structure, electrical conductivity, and permittivity holding particular importance. In electronic applications, niobium oxide, especially Nb_2_O_5_, the most thermodynamically stable niobium oxide, has garnered significant attention.

The dielectric constants for Nb_2_O_5_ have shown significant variation and inconsistency, ranging from 41 to 120. And its band gap spans from 3.4 to 5.3 eV [122], classifying Nb_2_O_5_ as an N-type semiconductor with a wide band gap. The conduction band is formed by the vacant 4d orbitals of Nb^5+^ and is approximately 0.2–0.4 eV greater than that of TiO_2_. [123]. Nb_2_O_5_ is frequently employed in synthesizing alkali metal niobates like MNbO_3_(M = Li, K, Na), commonly used in optoelectronic devices [124]. Due to its excellent dielectric properties, Ding et al. also applied it to Y_2_Ti_2_O_7_ microwave dielectric ceramics to improve its dielectric properties [125].

Nb_2_O_5_ exhibits varying bandgap energies (Eg) ranging from 3.1 eV (semiconducting behavior) to 5.3 eV (insulating behavior). The bandgap energy can be adjusted by adding foreign ions and other methods. The nanostructured Nb_2_O_5_ can significantly affect its electrical properties, potentially causing a blue shift in the band gap. According to Clima et al., different structures in sol-gel deposited Nb_2_O_5_ correspond to different permittivities, such as ortho, δA hexagonal, and δB hexagonal [126]. Simultaneously, their examination of the permittivity tensor reveals that Nb_2_O_5_ exhibits anisotropic dielectric behavior. 

When studying the dielectric constant (ε′) of Nb_2_O_5_, different structures yield different values [127]. For instance, amorphous Nb_2_O_5_ films formed via anodic oxidation can have dielectric constants ranging from 41 to 120. Meanwhile, sintered Nb_2_O_5_ pellets exhibit dielectric constants ranging from 38 to 165 depending on the processing routes [128]. Furthermore, Graca and colleagues [124] found that T-Nb_2_O_5_ and H-Nb_2_O_5_ powder compacts exhibit dielectric constant values of approximately 80 and 17, respectively, and a mixture of these phases reached ~600. Soares and colleagues [128] discovered that the dielectric constant of sintered Nb_2_O_5_ pellets differs based on the processing method. The electrical properties of bulk Nb_2_O_5_ are closely related to its crystal structure and material density, which are related to sintering conditions [124,128].

The electrical conductivity of niobium oxide is related to the formation of electrons in oxygen vacancies, with oxygen in the formation route determining conductivity [129]. Therefore, the electrical characteristics can significantly vary based on the experimental conditions. Under non-oxygen atmospheres, the conductivity of Nb_2_O_5_ increases owing to the creation of additional oxygen vacancies. When heated in air, however, the conductivity declines at ~550 K, attributed to molecular absorption reducing vacancies. Therefore, these alterations in transmission properties also relate to oxygen vacancies. This reliance on vacancies forms the foundation of the material’s technological applications.

In addition, electron mobility is an important parameter in the development of semiconductor devices, with studies indicating that the mobility of Nb_2_O_5_ increases with an increase in temperature. Indeed, the conductivity of Nb_2_O_5_ crystal exhibits an exponential dependence on temperature [130].

### 3.2. Optical Properties

Recently, there has been a growing interest and increasing scientific attention toward Nb_2_O_5_ due to its versatility as a multifaceted material. It is widely recognized as a transparent oxide semiconductor material [1,131,132], which exhibits transparency in the ultraviolet region owing to its wide band gap, insolubility in water, and stability in air. Due to the interesting characteristics of Nb_2_O_5_ films, including their wide band gap, high refractive index, excellent thermochemistry, and stability, they are currently used in several applications. These applications encompass, but are not limited to, photoelectric devices, catalysis, gas sensors, high-performance oxide glasses [133], and EC devices [106]. Nb_2_O_5_ has garnered attention due to its potential applications in solar cells, batteries, photodetectors, and other electronic devices [9,134].

Le and colleagues conducted studies to determine the band gap of crystalline Nb_2_O_5_ nanofibers, including H-Nb_2_O_5_, O-Nb_2_O_5,_ and M-Nb_2_O_5_, which were sintered at three different temperatures (773 K, 1073 K, and 1373 K). The band gaps for H-Nb_2_O_5_, O-Nb_2_O_5_, and M-Nb_2_O_5_ were measured at 3.85 eV, 3.77 eV, and 3.79 eV, respectively [135]. In a separate study, Abe reported the band gap values of orthotropic Nb_2_O_5_, synthesized via powder, to be 3.4 eV, while that for monoclinic Nb_2_O_5_ was 3.1 eV [136]. Furthermore, Abe studied how incorporating varying concentrations of Ge into monoclinic Nb_2_O_5_ films affected their band gap. The optical absorption edge of H-Nb_2_O_5_, without Ge doping, was measured at 3.1 eV. However, increased Ge concentration shifted the absorption edge to 3.35 eV [136]. During the early 2010s, Liu and colleagues utilized a two-step solution method to fabricate single-crystal porous Nb_2_O_5_ nanotubes. They also prepared homogeneous single-crystal Nb_2_O_5_ nanorods. The band gap values for Nb_2_O_5_ nanotubes and nanorods were 3.97 eV and 3.72 eV, respectively. The observed variation in band gap values, specifically the 0.25 eV difference between porous nanotubes and solid nanorods, can be attributed to the blue shift of the absorption edges in the former, caused by the quantum confinement effect in the hollow Nb_2_O_5_ nanotubes [137]. Brayner and colleagues similarly noticed a blue shift [138]. Agarwal and colleagues also investigated how grain size influences the electronic properties of Nb_2_O_5._ They proposed that the energy of the absorption edge and local coordination can be affected by variations in grain size [139].

As previously mentioned, Nb_2_O_5_ possesses a band gap ranging from 3.1 to 4.0 eV. Its optical properties are highly dependent on its crystallinity and grain morphology, allowing it to effectively absorb light in the UV and near-UV spectra or serve as a transparent material for ultraviolet light. Niobium oxide films exhibit electrochromism, transitioning from transparency to shades of brown, gray, or blue when Li^+^ or H^+^ plasma is introduced. Nb_2_O_5_ is capable of modulating its optical transmission state, switching between high transmittance (T ~ 85%) in a quasi-transparent state and low transmittance (<T ~ 10%) in the UV, visible, or near-infrared (IR) ranges. Moreover, the material’s coloration can vary between blue and brown, depending on its crystallinity structure [140].

The study of Nb_2_O_5_’s electrochromic properties dates back to 1980, when Reichman and Bard reported its excellent chemical stability and resistance to acid and alkali corrosion. These characteristics have prompted extensive research into its application as an electrochromic material. This electrochromic phenomenon is related to the continuous and reversible optical changes produced by electrochemistry, which macroscopically manifest as color changes. However, electrochromic materials are typically evaluated based on color rendering efficiency, encompassing transmittance contrast, response time, and chemical stability when transitioning from colored to bleached states under an electric field and charge injection. Thus, the material properties of Nb_2_O_5_ films play a significant role in determining these characteristics. Nb_2_O_5_ thin sheets exhibit a low refractive index (reported value of ~2–2.3) [141], with the degree of crystallinity influencing the refractive index, which can decrease from 2.30 to 2.20 when the material is subjected to heat treatment at 700 °C under ambient conditions [97].

### 3.3. Mechanical Properties

In addition to its optical and electrical properties, the mechanical strength of Nb_2_O_5_ plays a pivotal role in producing electronic devices, particularly in manufacturing actuators and flexible mechanical components. Thin films often experience stresses and strains due to limitations imposed by the deposition method or substrate.

Sputtered Nb_2_O_5_ films typically exhibit an average hardness (H) ranging from 5.6 to 6.8 GPa, with a Young’s modulus (Er) reaching 117 to 268 GPa. These mechanical properties are influenced by the crystalline structure of the film [97,142]. The MIM capacitor, incorporating a sputtered Nb_2_O_5_ film, underwent the collapse radius test, a commonly employed bending test technique. The findings indicated that the device exhibited remarkable robustness and resilience, enduring repeated testing for up to 2500 times [143].

## 4. Application of Nb_2_O_5_

Over the past few decades, the excessive exploitation and utilization of fossil fuels by humans has led to resource depletion and significant environmental challenges. Therefore, new energy sources and technologies are needed to alleviate the pressure on energy sources and the environment. The replacement of conventional internal combustion engines with mobile energy storage systems is crucial for phasing out fossil fuels. For these applications, it is necessary to have high energy density energy storage materials [144].

Lithium-ion batteries (LIBs) and supercapacitors (SCs), the two main devices for storing electric energy, are vital to daily life. LIBs are widely used as the primary energy supply for handheld devices, such as mobile phones and laptops. Additionally, they have gained widespread adoption in electric vehicles, garnering significant global attention [145,146]. Moreover, due to the unique electrical properties of Nb_2_O_5_, extensive research has been conducted on its applications in LIBs and SCs. Researchers have achieved performance optimization in both LIBs and SCs by modifying the properties of Nb_2_O_5_ thin films. The various applications of Nb_2_O_5_ reported in the literature are described in the following sections.

### 4.1. Lithium-Ion Battery

Batteries and HSCs are crucial in electric vehicles, smartphones, and more, driving advancements in related industries. Developing sustainable and high-capacity anode materials is a key parameter of high-efficiency battery technology. Graphite (Gr) is the most widely utilized anode material in LIBs [147]. Carbon nanotubes and graphene also hold significance as anode materials [148]. However, these carbonaceous materials are limited by high production costs, complex, large-scale manufacturing processes, and unsustainable routes. 

Consequently, it is imperative to explore the development of novel anode materials that are environmentally friendly and cost-effective [149]. Such advancements are essential to ensuring the sustainable growth of battery technology [150]. This push toward developing affordable and sustainable alternatives highlights the need for ongoing research and innovation in this field [151].

Recently, there has been significant interest in utilizing niobium oxide-based materials and their composites in LIBs. This is due to the unique properties that render them particularly suitable for this application, including their quasi-two-dimensional networks, ion insertion/extraction, abundant REDOX chemistry, and excellent chemical stability. Therefore, this discussion delves into various aspects of Nb_2_O_5_ application, including their classification, advantages, disadvantages, and research progress.

The anode materials for LIBs are assigned to three primary classifications: alloy, conversion, and insertion types [152]. While the specific capacity of insertion-type electrode material is theoretically lower than the other two, it possesses many advantageous properties that make it an ideal anode material for LIBs. In particular, it exhibits excellent cycling stability and the ability to maintain high capacity under high power conditions, critical factors for determining the performance of a battery.

**Figure 7:**
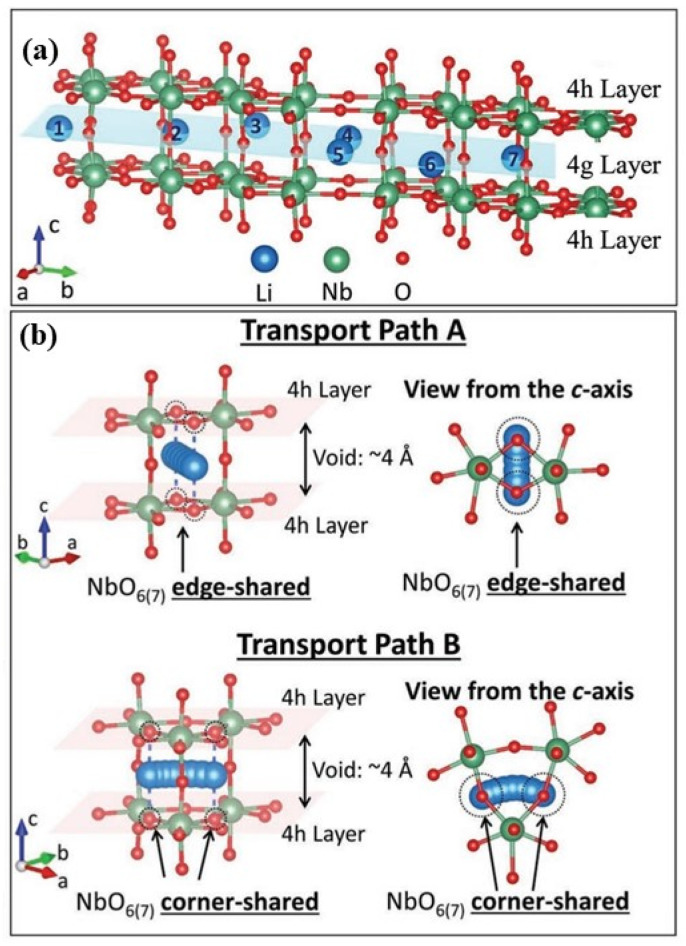
(**a**) Structure of lithiated T-Nb_2_O_5_ after geometry optimization. (**b**) Two kinds of Li-ion transport path topologies (Path A and Path B). Reprinted with permission from Ref. [36], Copyright 2017 American Chemical Society.

The insertion lithium storage mechanism exhibited by niobium (Nb)-based oxides positions them as highly promising candidates for replacing high-rate negative electrode materials [153]. The crystal structure of T-Nb_2_O_5_ comprises two alternating atomic layers, namely the loosely arranged 4g layer and the densely arranged 4h layer (Figure 7a). Due to the large atomic space, the 4g layer is a preferred storage and transport site for lithium ions. Theoretical calculations reveal that the lithium ions occupying the 4g layer are relatively stable. Based on the neighboring Nb-O bond structure in T-Nb_2_O_5_, two distinct Li-ion diffusion pathways exist: Path A and Path B (Figure 7b). These diffusion pathways offer direct transport channels, ample transport space, and low-energy barriers, facilitating rapid Li-ion transportation [36].

**Figure 8:**
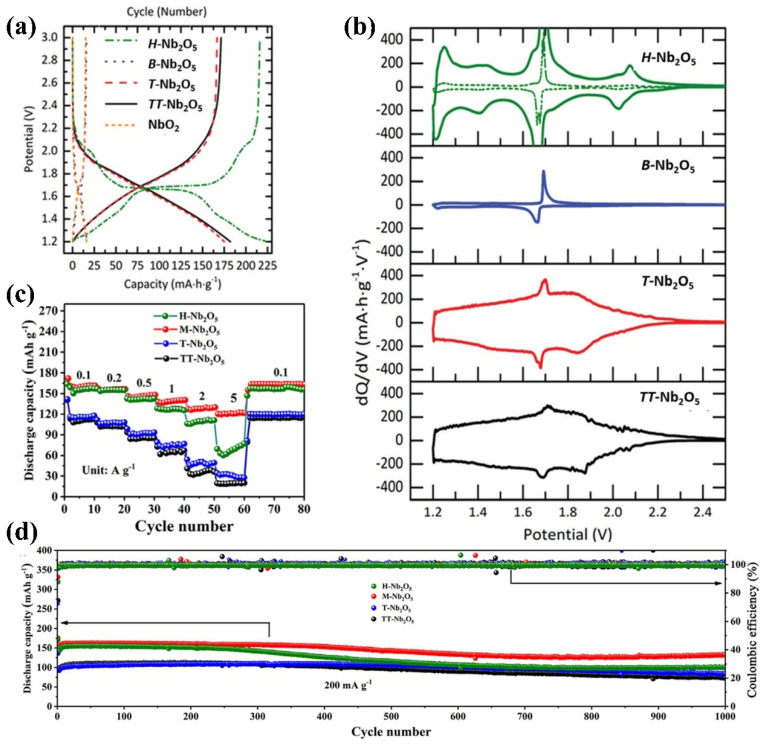
Electrochemical responses of the Nb_2_O_5_ polymorphs (H, B, M, T and TT). (**a**) Cycling performance tests at 1C (T, TT) or C/10 (B, H). (**b**) Differential capacity plots derived from the discharge/charge profiles. Reprinted with permission from Ref. [156], Copyright 2021 Royal Society of Chemistry. (**c**) Rate performances of Nb_2_O_5_ (H, M, T and TT) tested at various current densities ranging from 0.1, 0.2, 0.5, 1, 2, 5 and back to 0.1 A g^−1^. (**d**) Long cycling performances of Nb_2_O_5_ (H, M, T and TT) at a current density of 200 mA g^−1^. Reprinted with permission from Ref. [34], Copyright 2020 Elsevier.

First, most Nb-based oxides operate within a potential range from 1.0 to 2.0 V (relative to Li^+^/Li), with REDOX reactions involving Nb^5+^/Nb^4+^ and Nb^4+^/Nb^3+^ being the primary mechanisms. This operating potential range is selected to mitigate electrolyte deposition and the formation of lithium dendrites, critical for ensuring battery safety under high current densities and overcharging conditions. Second, compared to LTO, Nb oxides demonstrate a higher specific capacity, typically ranging from 200 to 400 mAh g^−1^. This enhances the appeal of Nb-based oxides as negative electrode materials for LIBs. Third, Nb oxide has a high Li^+^ diffusion coefficient, attributable to its unique structure, making it an attractive option as a negative electrode material in LIBs, as it can improve the battery’s overall performance [99,154]. Fourth, the low volume expansion rate of Nb oxide reduces the risk of structural damage caused by the high levels of expansion and contraction during lithium insertion. This property increases the stability and longevity of the battery (Figure 8) [155].

In addition, even at micron sizes, monoclinic Nb oxide possesses a remarkable energy storage capacity [22,157]. The findings from these studies provide strong evidence that Nb-based oxide materials are highly advantageous as alternative anodes in high-performance LIB applications. In particular, they boast suitable operating potentials, high specific capacities, high Li^+^ diffusion coefficients, and low volume expansion rates. Indeed, Nb-based oxides have the potential to play a major role in improving the performance and safety of LIBs for future energy storage applications.

#### 4.1.1. Lithium-Ion Battery Anode Performance

**Figure 9:**
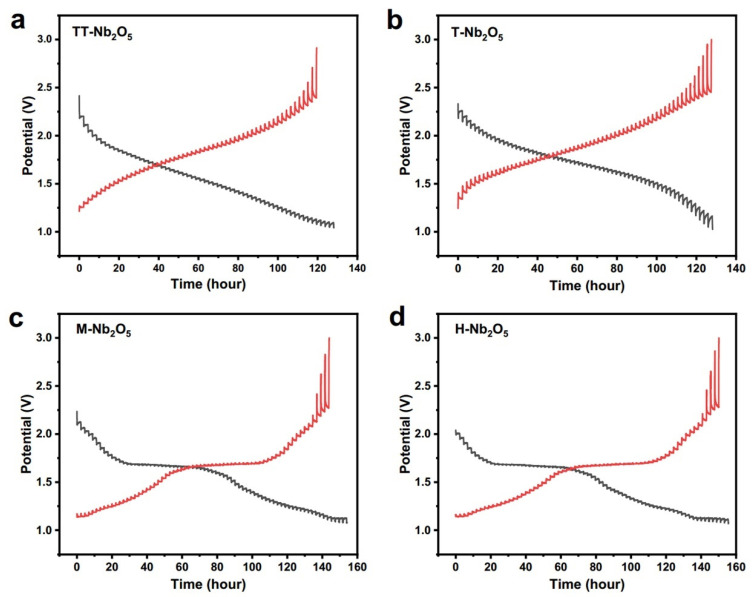
(**a**–**d**) GITT plots of TT-, T-, M- and H-Nb_2_O_5_, respectively. Reprinted with permission from Ref. [161], Copyright 2021 American Chemical Society.

There has been a recent increased focus on Nb-based oxide materials, particularly Nb_2_O_5_, as candidates for various energy storage applications. These materials have been thoroughly investigated for their potential utilization in technologies such as LIBs, SIBs, and SCs [158,159]. This heightened interest can be attributed to the unique set of advantages that these materials offer, including a high specific capacity, high Li^+^ diffusion coefficient (Figure 9), low volume expansion rate, and stable cycling performance. As such, Nb-based oxides are widely recognized as a promising avenue for developing advanced energy storage systems. In addition, the high operating voltage of Nb oxides generally exceeds 1.0 V vs. Li^+^/Li, representing a critical advantage that mitigates the decomposition of organic electrolytes and the development of solid–electrolyte–interface (SEI) films in batteries. These complications can arise from the instability of certain materials at lower voltages, leading to unwanted reactions or side effects that limit the performance and lifespan of the battery. Thus, the high operating voltage of Nb-based oxides can enhance the safety and efficiency of energy storage systems. Rapid energy storage and release are widely regarded as fundamental technologies for developing next-generation battery systems. Achieving fast energy storage performance can be realized through the use of advanced electrode materials, novel electrolyte formulations, or innovative cell designs. In particular, the utilization of Nb-based oxides has shown promise in enhancing the energy storage performance of batteries, owing to their high specific capacity, low volume expansion rate, and excellent cycling stability (Figure 8). By incorporating these materials into next-generation batteries, it is possible to achieve faster charging times, longer lifespans, and higher energy densities, ultimately paving the way toward more efficient and sustainable energy storage technologies [160].

Electrode active materials and their properties are crucial in influencing the overall performance of LIBs as they are essential in facilitating the reversible flow of ions during charging and discharging cycles [162]. In the early 1980s, Nb_2_O_5_ was first studied as a LIB material [163,164]. A fundamental discovery was made by Reichman et al., who reported that Nb_2_O_5_ could intercalate with Li^+^ ions, suggesting the possibility of reversible charging and discharging [163]. Kumagai’s group further explored the use of Nb_2_O_5_ as a cathode material for LIBs, employing advanced techniques, including XPS, XRD, and XAFS. Their investigations focused on characterizing the structural changes in the Nb_2_O_5_ cathode during charging and discharging and elucidating the underlying mechanisms governing its electrochemical behavior [165]. Among different crystal structures, tetragonal-Nb_2_O_5_ exhibits the most favorable cycling performance. In fact, it can achieve a discharge capacity of up to 190 mAh g^−1^ and sustain up to 30 cycles. XRD analysis revealed that the orthorhombic and tetragonal structures of Nb_2_O_5_ maintain their original lattice despite experiencing slight changes in cell volume following Li^+^ embedding. Meanwhile, the unique two-dimensional and layered structure of tetragonal Nb_2_O_5_ allows it to accommodate very high concentrations of embedded ions [165]. In 2011, researchers, led by Goodenough proposed TiNb_2_O_7_ as a notable material within the TiO_2_-Nb system, demonstrating its potential as a negative electrode material for LIBs [166]. In 2013, Dunn’s group reported that orthorhombic Nb_2_O_5_ (T-Nb_2_O_5_) exhibits exceptional electrochemical energy storage capabilities at high rates, primarily due to the utilization of Li^+^ embedded pseudocapacitance [28]. Following this discovery, Nb_2_O_5_-related anode materials regained significant attention due to their promising application prospects in fast-charge energy storage devices.

Research on Nb_2_O_5_ LIBs has also included exploring various electrolytes and electrode compositions with varying amounts of graphite. Reducing the graphite content within the electrode composition leads to a decrease in discharge capacity. Furthermore, researchers have explored the application of sputtered Nb_2_O_5_ as a LIB electrode. For example, Nakazawa and colleagues reported a battery featuring a sputtered Nb_2_O_5_ negative electrode with a thickness of 100 nm, achieving highly favorable charging and discharging performance, maintaining a capacity range of 310–380 mA h cm^−3^ over 500 cycles [167].

#### 4.1.2. Lithium Storage Mechanism

The electrochemical behavior of Nb_2_O_5_ electrode materials, which exhibit distinct crystal structures resulting from variations in temperature and pH value, demonstrates noticeable differences. These distinctions can be attributed to the specific material processing method employed and the underlying principles of the fuzzy reaction. Further understanding of the reaction mechanism can be obtained by exploring the storage of Li^+^. Bard et al. conducted the primary investigation on the electrochemical behavior of Li^+^ in Nb_2_O_5_ [101]. They found that the Nb_2_O_5_ electrode exhibited electrical conductivity in an acetonitrile solution containing 0.8 M LiClO_4_, and an electrochemical redox process occurred between Nb_2_O_5_ and Li^+^. Hence, Li^+^ could undergo a reversible reaction with Nb_2_O_5_, resulting in the formation of Li_x_Nb_2_O_5_. The redox reaction mechanism can be represented by the following equation:

(1) $${Nb}_{2}O_{5}+xLi++xe^{-}↔Li_{x}{Nb}_{2}O_{5}\left(x=0–2\right)$$

When x = 2, it corresponds to a maximum theoretical capacity of 200 mA h g^−1^.

Typically, the theoretical capacity of Nb_2_O_5_ is determined using Equation (2), which is derived from the two-electron redox reaction, as follows:

(2) $$Q_{theoretical}=\frac{nF}{M_{W}}=\frac{2\times 96485.3\;C\;{mol}^{-1}}{265.8\;g\;{mol}^{-1}}=725.9\;C\;g^{-1}=201.6\;mA\;h\;g^{-1}$$

where “n” represents the number of electrons involved in the reaction, “F” denotes the Faraday’s constant, “Mw” signifies the molecular weight of the active material, and 3.6 serves as the conversion factor between coulombs (C) and conventional milliampere-hours (mA h).

Among the various configurations of Nb_2_O_5_, the pseudo-hexagonal system TT-Nb_2_O_5_, orthorhombic system T/O-Nb_2_O_5_, and the monoclinic system H-Nb_2_O_5_ have undergone extensive investigation as anode materials for energy storage systems, each with distinct mechanisms for storing lithium. In 1999, Kumagai and colleagues [168] evaluated the electrochemical behavior of three different Nb_2_O_5_ configurations obtained at different heating temperatures. These configurations were designated the hexagonal, orthogonal, and monoclinic phases. The heating temperature during the synthesis process significantly impacted the crystal structure and properties of Nb_2_O_5_. They obtained Nb_2_O_5_ samples with different crystal structures by controlling the heating temperature. The Nb_2_O_5_ compounds exhibited comparable electrical activity, demonstrating discharge capacities ranging from 160 to 180 mA h g^−1^ within the potential range of 1.2 to 3.0 V (vs Li^+^/Li).

**Figure 10:**
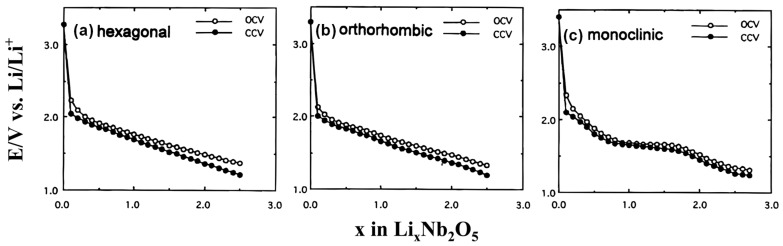
Initial discharge curves of (**a**) hexagonal, (**b**) orthorhombic, and (**c**) monoclinic Nb_2_O_5_ powder pressed electrodes as a function of x in Li_x_Nb_2_O_5_ [168].

Hexagonal and orthorhombic Nb_2_O_5_ electrodes do not exhibit distinct potential plateaus during charging and discharging. Instead, the potential varies linearly with the x value of Li_x_Nb_2_O_5_ (Figure 10a,b). On the contrary, the charge–discharge curve of the monoclinic Nb_2_O_5_ electrode displays multiple potential platforms (Figure 10c). Based on the research findings, the hexagonal and orthorhombic Nb_2_O_5_ undergo a single ternary phase formation of Li_x_Nb_2_O_5_ while embedding Li^+^. Meanwhile, the lithiation process of monoclinic Nb_2_O_5_ involves two-phase reactions, transitioning from x = 0.8 to x = 1.8 within Li_x_Nb_2_O_5._

The crystal structure of T-Nb_2_O_5_, characterized by its NbO_6_ and NbO_7_ polyhedra and the irregular arrangement of Nb and O ions, significantly influences its properties [28], as discussed in Section 2.1.1. Chen et al. [36] investigated the rapid lithium storage mechanism of T-Nb_2_O_5_ by combining theoretical calculations and experimental measurements. The T-Nb_2_O_5_ structure consists of alternating atomic layers that exhibit two distinct arrangements. Approximately 40% of the O^2−^ ions occupy the 4g Wyckoff site, resulting in relatively loosely packed 4g layers. The remaining ions occupy the 4h Wyckoff site, forming more densely packed 4h layers. All niobium ions are situated in the relatively dense 4h layer, while lithium ions are positioned in the loosely packed 4g layer, providing space to accommodate Li^+^ ions, while the dense 4h layer facilitates the formation of a well-defined bridge coordination between oxygen and Li^+^ ions. This unique arrangement of bridging sites enables the formation of rapid Li^+^ diffusion paths with low migration barriers [36].

Kodama and colleagues demonstrated that a continuous transition from Nb^5+^ to Nb^4+^ occurs during the insertion of Li^+^ ions. Additionally, the cell volume of orthorhombic Nb_2_O_5_ undergoes only slight changes after the inclusion of Li^+^ ions [165]. According to Dunn et al., the preferential insertion of Li^+^ ions occurs through the (180) and (001) planes in T-Nb_2_O_5_. Furthermore, the redox reaction between Li^+^ ions and T-Nb_2_O_5_ occurs within a single-phase system [169], where the {001} group planes form a channel conducive to Li^+^ migration. Therefore, the orthorhombic T-Nb_2_O_5_ system boasts a unique crystal structure that features a two-dimensional Li^+^ channel unaffected by Li^+^ reincarceration behavior. This facilitates rapid Li removal in T-Nb_2_O_5_. Furthermore, theoretical calculations indicate that the (001) surface of T-Nb_2_O_5_ facilitates the transport of ions along degenerate paths with low energy barriers due to the large oxygen–oxygen distance of 3.9 Å [168,170]. In addition, the unique “room-and-pillar” NbO_6_/NbO_7_ framework of T-Nb_2_O_5_ offers a stable structure for embedding Li^+^ ions, ensuring that no phase transitions occur during the ion insertion process [22]. Nb_2_O_5_‘s special layered structure allows bulk T-Nb_2_O_5_ to facilitate rapid Li^+^ diffusion, exhibiting excellent intercalation pseudocapacitance [165].

T-Nb_2_O_5_ and TT-Nb_2_O_5_ are considered pseudocapacitor materials due to their similar crystal structure [155]. However, as TT-Nb_2_O_5_ possesses a more disordered crystal structure, it encounters more structural obstacles during Li^+^ embedding [171]. T-Nb_2_O_5_ exhibits a higher reversible specific capacity than TT-Nb_2_O_5_, leading to a noticeable difference in their performance.

The lithium storage mechanism of H-Nb_2_O_5_ differs from that of T-Nb_2_O_5_ and TT-Nb_2_O_5_, showcasing distinct characteristics in their respective behaviors. In contrast to T-Nb_2_O_5_ and TT-Nb_2_O_5_, monoclinic H-Nb_2_O_5_ features a densely packed oxygen array and exhibits a more significant repulsion intercalation effect. As previously mentioned, the extensive intercalation of lithium within the H-Nb_2_O_5_ structure can result in phase separation [168]. This phenomenon is generally recognized as the primary factor contributing to the inferior rate performance observed in numerous studies when comparing H-Nb_2_O_5_ to T-Nb_2_O_5_. However, Ding et al. [160] found that the performance failure of H-Nb_2_O_5_ was attributable to the anisotropy of electron and ion conduction within the H-Nb_2_O_5_ crystals. This asynchronous phase transition during lithium (de)intercalation is a direct consequence of the phase separation, further impacting the performance of H-Nb_2_O_5_ compared to T-Nb_2_O_5_ [172]. Researchers have employed a strategy involving the application of uniform amorphous carbon shells as a coating on the surface of micron-scale single-crystal H-Nb_2_O_5_ particles. This serves to homogenize electron and Li^+^ transport. This optimization technique significantly enhanced the fast lithium storage performance of the negative electrode composed of H-Nb_2_O_5_, surpassing the performance of many reported negative electrodes based on LTO and T-Nb_2_O_5_.

In conclusion, the inherent structural advantages of Nb_2_O_5_ have attracted considerable attention in advanced electrochemical energy storage applications. For example, the high operating voltage of Nb_2_O_5_ (>1.0 V vs. Li^+^/Li) can inhibit the decomposition of the electrolyte and the formation of SEI film and lithium dendrites, ensuring the safety of the battery. Continuous and high-demand research efforts will be focused on exploring more effective strategies for enhancing the conductivity of niobium-based oxides. Structural optimization, surface engineering, and carbon modification remain the primary avenues for investigating this field. Meanwhile, the composite utilization of anode electrode materials with high theoretical capacity and synergistic effects is significant.

#### 4.1.3. Effect of Nb_2_O_5_ Nanostructures

Zero-dimensional (0D) Nb_2_O_5_ nanostructures, specifically large-sized nanoscale particles, have been the subject of recent studies. These investigations have primarily focused on exploring the unique properties and applications of such nanostructures. However, it is worth noting that the repetitive usage of these large-sized 0D Nb_2_O_5_ particles may lead to a decrease in performance or capacity due to potential degradation or other cycling-related factors [173,174,175,176]. The uniform distribution of particles within a battery is essential as it provides a large surface area and abundant reaction sites. This prevents particle agglomeration during the charging and discharging process, ultimately improving the electrochemical performance of the battery. The amorphous Nb_2_O_5_ precursors prepared in the air by the sol-gel method were calcined at different temperatures to obtain different crystalline phases of Nb_2_O_5_ (amorphous, T, TT). The grain size of these phases (hexagonal, orthorhombic, and monoclinic) increases with rising temperature. Extensive testing has demonstrated that Nb_2_O_5_ exhibits remarkable cyclic stability. In addition, Liu and colleagues [175] synthesized T-Nb_2_O_5−x_ particles using a straightforward sol-gel process, followed by post-etching and calcination. However, a small portion of cell recombination occurred due to the removal of Sr and Ca atoms during the acid etching process. The significant stress associated with this process leads to the formation of high-density defects, contributing to the generation of more reaction sites so that the final sample has good magnification performance and cycle stability. Chen et al. conducted a solvothermal method to obtain Nb_2_O_5_ nanomaterials with varying graphene contents (1, 2, 3, 4, and 5 wt.%) and superphosphorus as conductors [176].

One-dimensional Nb_2_O_5_ nanostructure: Using pure Nb_2_O_5_ with one-dimensional nanostructures as an anode material for LIBs is not widespread. This may be attributed to the intricate preparation requirements and challenging control of the associated processes. Most research efforts in one-dimensional nanostructures have predominantly focused on nanofibers [52] and nanorods [48]. Despite the challenges associated with their preparation, the distinctive aspect ratio of pure Nb_2_O_5_ in one-dimensional nanostructures offers an effective pathway for transmitting Li^+^ ions. This unique characteristic continues to garner significant interest within the research community. In some instances, regulating heating rates during the heat treatment is considered an effective approach to fabricating nanofibers with exceptional properties.

Two-dimensional Nb_2_O_5_ nanostructure: Generally, two-dimensional Nb_2_O_5_ nanomaterials used for LIBs include nano-thin films [177], nano-sheets [178,179] and nanoribbons [180,181]. One advantage of controlling the heating rate during the treatment process is its ability to create a continuous framework that facilitates efficient ion and electron diffusion; more Li^+^ storage sites are on the open edge. Consequently, by optimizing reaction kinetics, controlling the heating rate can enhance the overall performance of these materials. Furthermore, the anisotropic growth of two-dimensional nanomaterials offers additional opportunities for REDOX reactions in batteries. Nano-thin film electrode materials can be prepared through physical or chemical deposition methods. Due to the large energy density area, two-dimensional flexible nanofilms grown directly on the substrate without additives and conductive agents can be used in micro-energy storage devices.

In the study conducted by Zhou et al. [182], metallic Nb powder served as a precursor for synthesizing Nb_2_O_5_ nanoribbons. Subsequent investigation primarily focused on evaluating the electrochemical characteristics of these nanoribbons when employed as anode materials in LIBs. Liu et al. successfully fabricated Nb_2_O_5_ nanosheets with dimensions of 50 nm in thickness and 500 nm in length through a hydrothermal reaction [178]. The excellent rapid energy storage capacity of these two-dimensional Nb_2_O_5_ nanostructures can be attributed to their large active surface area and short Li^+^ diffusion distance.

**Figure 11:**
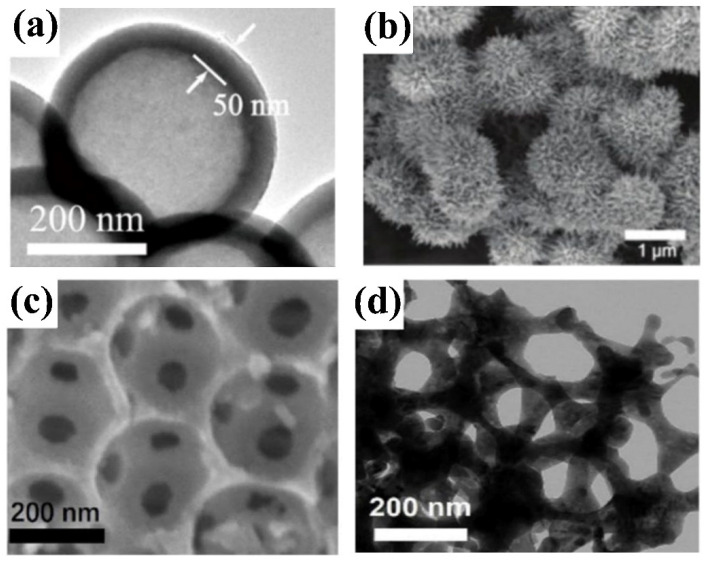
(**a**)TEM image of HM-Nb_2_O_5_ collected 20 min after reaction. Reprinted with permission from Ref. [183], Copyright 2018 American Chemical Society (**b**) Field emission SEM image of Nb_2_O_5_ microspheres. Reprinted with permission from Ref. [186], Copyright 2007 Royal Society of Chemistry. (**c**) SEM image and (**d**) TEM image of 3DOM T-Nb_2_O_5_. Reprinted with permission from Ref. [188], Copyright 2017 Elsevier.

In addition to the zero-dimensional (0D), one-dimensional (1D), and two-dimensional (2D) structures, Nb_2_O_5_ exhibits various heterogeneous multi-level complex structures. Notable examples include hollow porous spheres [183], sea urchin-shaped spheres [184,185,186], micro-structures composed of nanoparticles [187], 3D pore frames [188,189], and self-assembled nanosheets [190]. The casting process employed to generate complex structures, such as hollow porous spheres in Nb_2_O_5_, offers several advantages that compensate for the specific advantages that individual structures alone cannot achieve. The casting method allows for precise control over the structure’s shape, size, and composition, enabling enhanced properties such as improved structural stability, increased surface area, and optimized electrolyte penetration. The synergy created by incorporating various structures within a single material can lead to superior performance in various applications. Indeed, complex microsphere structures in Nb_2_O_5_ can be synthesized using relatively straightforward methods, such as precipitation, hydrothermal, and solvothermal techniques. Sun et al. [183] showed that a simple oil bath process can also generate nanostructures. They designed a technique for preparing hollow mesoporous Nb_2_O_5_ (HM-Nb_2_O_5_) nanospheres with a diameter of ~300 nm by calcination at 600 °C. The thickness of the nanosphere shell was achieved by extending the reaction time caused by the controlled hydrolysis of urea (Figure 11a). Liu et al. successfully synthesized sea urchin Nb_2_O_5_ microspheres with a 20 nm diameter in a glycerol-isopropyl alcohol mixture using a simple solvothermal method through the self-assembly of nanorods (Figure 11b) [187]. Lou et al. [188] achieved successful synthesis of three-dimensionally ordered macroporous (3DOM) T-Nb_2_O_5_ nanostructures using self-assembled polystyrene (PS) microsphere colloidal crystals as a hard template. The process involved immersing the template in a precursor solution to form the desired T-Nb_2_O_5_ nanostructures. This approach yielded a substantial quantity of tightly packed macropores measuring 200 nm in diameter. The surface of the synthesized material exhibited many additional macropores, ranging from 60 to 130 nm, along with mesoporous structures (as illustrated in Figure 11c,d).

**Figure 12:**
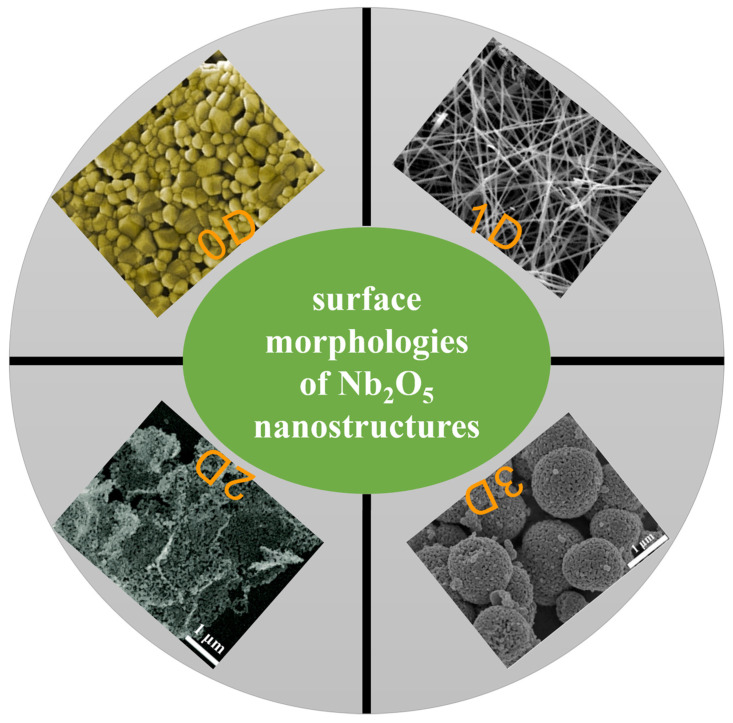
Classification of Nb_2_O_5_ nanostructure surface morphologies. The pictures are from the literature.

Overall, the 0D nanostructure offers a significant specific surface area that enhances the contribution of pseudocapacitors. However, it is limited by particle agglomeration resulting from repeated high-rate charging and discharging, which negatively impacts electrode lifespan, contradicting its intended purpose. In the case of 1D nanomaterials grown along (001) surfaces, their unique aspect ratio is crucial in facilitating the rapid transmission of Li^+^ ions, significantly enhancing Li storage dynamics. Nevertheless, the inherent stable chemical properties of Nb_2_O_5_ contribute to the complexity and lack of control observed during the preparation process. Generally, 2D nanostructures with expandable anisotropic ion shuttle paths and open edges offer larger Li attachment sites, enabling higher capacity storage in LIBs. Researchers favor complex nanostructures, including heterogeneous multistage mesoporous structures, due to their inherent versatility and ability to address specific requirements that cannot be met by a single structure alone. This versatility extends to promoting solvent diffusion and fulfilling diverse application needs. However, complex nanostructures are also constrained by their structural durability deficiencies, resulting in a low volume packing density. Therefore, an increasingly popular approach to address these limitations involves optimizing structural engineering through the formation of composite materials (Figure 12).

Conclusion: Nb oxide plays a significant role in energy storage thanks to its distinctive crystal structure, similar to other important materials, and its exceptional chemical stability. Among its various forms, Nb_2_O_5_ offers clear advantages over commercial graphite anode materials. Nb_2_O_5_ boasts high power, enhanced safety, facilitation of abundant REDOX reactions, and minimal volume expansion. These advantages contribute to its potential for commercial applications, particularly in small batteries and the intercalated pseudocapacitor lithium storage mechanism. However, it is essential to acknowledge that while employing a high-voltage window can prevent the formation of lithium dendrites, it can also lead to a trade-off in terms of lower energy density. As a result, Nb_2_O_5_ faces many challenges in its electrochemistry application due to its low conductivity as a semiconductor material and harsh synthesis conditions.

### 4.2. Supercapacitor

Electric vehicles are witnessing a significant shift toward using batteries and HSCs, with applications in consumer electronics, large power stations, and hybrid electric vehicles (HEVs) [191,192]. SCs offer distinct advantages compared to batteries, including significantly higher power density, longer cycle life, and rapid charging capabilities. However, the primary disadvantage of SCs lies in their relatively low energy density due to limitations in electrode size, restricting their storage capacity. One potential strategy to address these challenges is to focus on developing high-energy-density electrodes.

The primary benefits of Nb_2_O_5_-based material being used in SCs and LIBs can be summarized as follows: (1) Nb_2_O_5_-based materials will not precipitate metal Li due to their high potential platform; (2) Nb_2_O_5_-based materials are non-toxic with higher specific capacity than Li_4_Ti_5_O_12_; (3) Nb_2_O_5_-based material exhibit high thermodynamic stability, ensuring good safety performance of LIBs; (4) Nb_2_O_5_-based materials feature fast electrochemical reaction kinetics and long cycle life.

However, it has a drawback in its inherent poor electronic conductivity. In addition, Nb_2_O_5_-based materials lack a flat charge-discharge platform and possess a lower theoretical capacity than other alloy-type or converted compounds [153,193]. Furthermore, a power capacity imbalance exists between fast non-Faraday capacitive cathodes and slow Faraday battery-type anodes [30]. Despite these limitations, Nb_2_O_5_-based materials hold promise in EESC devices due to their unique structural advantages, strong rate performance, and cycle stability.

#### 4.2.1. High Rate Electrode

Kim et al. conducted a study to assess the impact of crystallinity on the capacitance response of Nb_2_O_5_ for SCs [171]. Meanwhile, Augustyn and colleagues demonstrated the utilization of electrodes with a thickness of up to 40 mm to achieve high-speed charge storage devices through intercalated pseudocapacitors [28]. Lubimtsev et al. conducted an additional study to investigate the underlying factors contributing to the high-rate behavior of intercalated pseudocapacitors in Nb_2_O_5_ crystals [194]. An effective approach to address these challenges to developing high-energy-density electrodes is combining conductive carbon materials, doping techniques, and morphology control in the design of composite materials [195].

**Figure 13:**
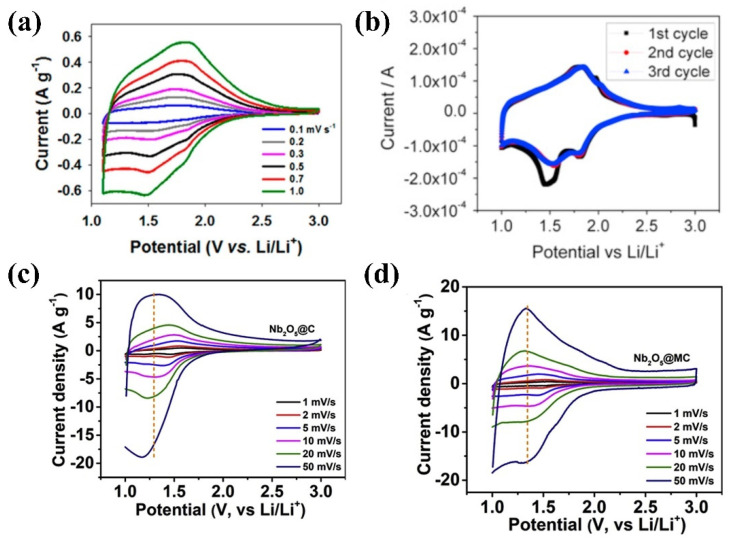
(**a**) CV curves of T-Nb_2_O_5_@C NCs at various sweep rates from 0.1 to 1.0 mV s^−1^. Reprinted with permission from Ref. [30], Copyright 2015 American Chemical Society. (**b**) Cyclic voltammetry curves of sample C-T-Nb_2_O_5_ for first three cycles at a scan rate of 0.1 mV s^−1^. Reprinted with permission from Ref. [197], Copyright 2014 Elsevier Cycle voltammetry curves: (**c**) Nb_2_O_5_@C and (**d**) Nb_2_O_5_@MC. Reprinted with permission from Ref. [199], Copyright 2018 Elsevier.

Lim and colleagues [30] employed a general process to prepare core-shell T-Nb_2_O_5_@carbon nanocrystals (T-Nb_2_O_5_/C NCs) and TT-Nb_2_O_5_/C composite materials. In a study by Lim et al. [196] the anode was fabricated using mesoporous T-Nb_2_O_5_@carbon nanocomposites. The resulting HSCs, assembled with activated carbon (AC) as the cathode, exhibited an impressive energy density of 74 Wh kg^−1^ and a power density of 18.51 kW kg^−1^ (Figure 13a). After subjecting the HSCs to 1000 cycles within the voltage range of 1–3.5 V at a current density of 1 A g^−1^, the capacity retention rate remained approximately 90%. This indicates that the electrode material exhibited commendable cycling stability and retained a significant portion of its initial capacity even after prolonged use. In another study, Wang et al. [197] utilized thin carbon-coated T-Nb_2_O_5_ nanowires to assemble 3 V T-Nb_2_O_5_@C||AC HSCs. The HSCs exhibited a prominent energy density of ~43 Wh kg^−1^ at 7.5 kW kg^−1^ and significant cycle stability (Figure 13b). Wang and colleagues developed an SC electrode comprising a closely mixed network of carbon nanotubes (CNTs) and Nb_2_O_5_ nanocrystals. This innovative electrode design aimed to achieve SCs with high capacitance, excellent rate performance, and cycling capability [198]. Furthermore, Zhang and colleagues [199] successfully prepared T-Nb_2_O_5_@carbon (Nb_2_O_5_@C) and T-Nb_2_O_5_@mesoporous carbon (Nb_2_O_5_@MC). Both samples were coated with porous carbon shells, enhancing structural stability and electrochemical performance (Figure 13c,d). The improved electrochemical performance of Nb_2_O_5_@MC can be attributed to the Nb_2_O_5_ nanoparticles with a high specific surface area (SSA), enabling more charge storage capacity. The interlinked mesoporous carbon shells also facilitate efficient ion diffusion and enhance charge transfer kinetics within the electrode material.

Recently, researchers have been exploring using metal-organic frameworks (MOFs) as a promising platform for preparing porous carbon materials and porous tetramethyl orthosilicate (TMOs). MOFs are renowned for their high porosity, large specific surface area, and well-defined porous structures. These properties make MOFs an attractive choice for creating materials with desirable porosity and surface area, with applications in diverse fields, including energy storage and catalysis. For example, Liu et al. [200] prepared T-Nb_2_O_5_ quantum dots (QDs) encapsulated in N-doped porous carbon derived from ZIF-8, referred to as NQD-NC. The resulting T-Nb_2_O_5_ QD was a unique composite material with enhanced properties. Indeed, the design of carbon nanotube (CNT) composites has emerged as an effective strategy to enhance the electrochemical performance of electrode materials. CNTs possess high electron conductivity and a large specific surface area, making them ideal for improving the performance across various electrochemical reactions. The high electron conductivity of CNTs facilitates the rapid movement of electrons within the composite, enabling efficient transfer during electrochemical processes. This promotes faster reaction kinetics and can lead to enhanced electrochemical performance [201]. For instance, Wang [202] and colleagues prepared T-Nb_2_O_5_ nanocrystals grown in situ on carbon nanotubes. Graphene, known for its substantial specific surface area, impressive flexibility, superconductivity, and wide voltage window, is being extensively investigated as a potential electrode material for SCs [203]. The doping process can be utilized to improve the electronic conductivity of materials by reducing the band gap, a feature particularly crucial in the context of SCs [204,205].

In summary, the challenge of low electrical conductivity in Nb_2_O_5_ can be effectively addressed through two common strategies: fabricating nanostructures with diverse morphologies and integrating Nb_2_O_5_ with carbon-based materials. These approaches have demonstrated significant effectiveness in overcoming the conductivity limitations associated with Nb_2_O_5_.

#### 4.2.2. Hybrid Supercapacitors

In contrast to rechargeable batteries, SCs exhibit an extended operational lifespan and capacity to facilitate rapid charge and discharge processes. These qualities contribute to their high power density and long cycle life. However, SCs typically possess a lower energy density than rechargeable batteries. Meanwhile, LIBs are known for their high energy density, providing significant stored energy. However, they are often limited in power density and cycle life. A hybrid supercapacitor (HSC) comprising a positive metal oxide electrode with pseudocapacitance behavior and a negative activated carbon electrode with EDLC behavior is an effective method to improve the energy density of EDLC.

The operation principle of this HSC involves reversible non-Faraday reactions, such as the adsorption of Li^+^ and Na^+^ ions on the surface of active and porous carbon materials at the positive electrode, facilitating charge storage. Simultaneously, reversible Faraday reactions take place at the metal oxide electrodes. SCs adopting this asymmetric configuration can accumulate charge utilizing the Faraday REDOX electrochemical reaction process. This enhances the specific capacitance and extends the operating voltage range, ultimately improving the energy density of the hybrid capacitor.

Previously, extensive research on RuO_2_ as an electrode material for HSCs has been primarily driven by its excellent specific capacitance, cyclic stability, and conductivity [206]. Nonetheless, the adoption of RuO_2_ as an electrode material for HSCs is hindered by its high cost, which restricts its widespread application.

Recent studies have revealed that bulk Nb_2_O_5_ has a higher capacity than the conventional Li_4_Ti_5_O_12_ under high magnification conditions. This suggests that Nb_2_O_5_ exhibits pseudocapacitive electrochemical characteristics. This phenomenon is explicitly observed on the surface of Nb_2_O_5_ rather than throughout the entire bulk crystal.

DFT calculations have indicated that forming a solid solution by incorporating lithium atoms at specific sites enables the selective provision of electrons to adjacent atoms. This results in the reduction of niobium within that region. The observed high specific capacitance in the case of Nb_2_O_5_ may be attributed to its nanoparticle structure. The presence of nanoparticles provides a larger surface area for electrochemical reactions, resulting in enhanced pseudocapacitance and overall improved performance at high magnification [194]. These results also provide directions for improving the performance of nanoporous Nb_2_O_5_.

Among the various crystal structures of Nb_2_O_5_, orthogonal Nb_2_O_5_ exhibits the highest relative capacity. However, although the nanoparticle structure of Nb_2_O_5_ contributes to its exceptional performance at high magnifications, the formation of this structure at temperatures above 600 °C can potentially lead to nanoparticle aggregation. Nano-scale rhombic system Nb_2_O_5_ is considered a favorable material for enhancing anode system dynamics. Despite the challenges associated with synthesizing small-sized rhombic Nb_2_O_5_ nanoparticles, alternative approaches have been developed to incorporate Nb_2_O_5_ into nanocomposite structures. One example is synthesizing mesoporous Nb_2_O_5_/carbon (m-Nb_2_O_5_-C) nanocomposites using a one-pot method assisted by block copolymer self-assembly [196]. Nb_2_O_5_@C NCs with controllable crystal phases, including the rhombic system (T) and pseudohexagonal system (TT), were synthesized using a one-pot microemulsion method. The pH condition of the water-in-oil microemulsion system significantly influences the control of the crystalline phase of Nb_2_O_5_ [30]. Under acidic conditions, T-Nb_2_O_5_@C nanocrystals are formed, while under alkaline conditions, TT-Nb_2_O_5_@C nanocrystals are obtained.

Therefore, utilizing Nb_2_O_5_ as an SC electrode and combining it with conductive carbon materials to improve charge transfer has emerged as a new research direction. For example, materials such as T-Nb_2_O_5_/graphene and Nb_2_O_5_/CNTs have demonstrated remarkable power density and cycling performance [198,202,207,208].

In addition, selecting suitable electrolyte solutions presents a challenge when employing Nb_2_O_5_ for SC applications. Recent research has indicated that using an electrolyte consisting of 1 M lithium perchlorate in a mixture of ethyl carbonate and dimethyl carbonate leads to the attainment of the highest capacitance and Coulomb efficiencies [208]. Moreover, although the Nb_2_O_5_ electrode has excellent lithium storage performance, due to the lack of lithium resources and the low cost and abundant sodium resources, the application of sodium ion batteries (SIB) has been widely studied recently. Meanwhile, the application of its energy storage performance in SIB has declined sharply due to its larger Na-ion radius and sluggish Na-ion diffusion. Therefore, preparing new Nb_2_O_5_ electrodes using various modification strategies to improve the electrochemical performance of SIB is an important research direction.

## 5. Conclusions and Perspectives

This review provided an overview of the fundamental crystal structure and physical and chemical properties of Nb_2_O_5_. Various synthesis methods for fabricating Nb_2_O_5_ polycrystals and the general applications of Nb_2_O_5_ in lithium batteries and supercapacitors (SCs) were summarized.

Nb_2_O_5_ exhibits polymorphs, including pseudohexagonal (TT-Nb_2_O_5_), orthorhombic (T-Nb_2_O_5_), and monoclinic (H-Nb_2_O_5_) etc. The crystal structures of these different phases were presented, and the research progress pertaining to their crystal structures and applications was discussed. The TT phase Nb_2_O_5_ was given particular emphasis. Various nanostructured Nb_2_O_5_, including nanopores, nanosheets, nanorods, nanochannels, and nanowires, have been successfully synthesized using different methods. However, it’s worth noting that there are still unexplored and uncharacterized nanoforms.

Similar to many other transition metal materials, pure Nb_2_O_5_ primarily exhibits poor conductivity. To address this limitation, incorporating heteroatoms, carbon, metals, and conductive polymers has proven effective in enhancing the electrical conductivity of Nb_2_O_5_. Research on forming oxygen vacancies by doping non-metallic elements in Nb_2_O_5_ remains relatively limited. Further investigations in this area are necessary to improve the electrochemical performance of such materials.

Nb_2_O_5_ plays an essential role in energy storage due to its unique crystal structure for fast charging, rich REDOX reaction capability, intercalation-based storage mechanism, high chemical stability, ultra-small volume expansion, and high potential window, which often served as electrode material for Li-ion batteries or supercapacitors. However, the electrochemical performance of Nb_2_O_5_-based electrodes still faces two key challenges. The first challenge is that Nb_2_O_5_ exhibits low conductivity and a high synthesis temperature. The second challenge is that Nb_2_O_5_ has a relatively low theoretical capacity and energy density. The T-Nb_2_O_5_ has fast rate capability with limited space for cations intercalation, while H-Nb_2_O_5_ offers more cation intercalation sites with slow rate capability. Active particles are frequently reduced to nanoscale dimensions to overcome the relatively slow solid-state ion diffusion and achieve rapid charging and high power. Optimizing the crystal phase or nanostructure of Nb_2_O_5_ is a promising approach to enhancing both rate and capacity performances. Another effective strategy involves doping other materials with Nb_2_O_5_.

In addition, Nb_2_O_5_ has been utilized as a sensing material for sensors. The synergistic integration of Nb_2_O_5_ with biomedicine has been explored to develop innovative solutions for diagnostics, therapeutics, and medical devices. Recently, Nb_2_O_5_ has been investigated for application in transistors, memristors, and superconductors. A better understanding of the Nb_2_O_5_ structure–property relationship is needed for further research efforts. This will facilitate the full harnessing of its capabilities and optimize its performance.

## Figures and Tables

**Table 1:** Classification of Nb_2_O_5_ polymorphs.

Polymorphism	Designation of the Nb_2_O_5_ Form	Crystallization Temperature (K)	Ref.
TT-Nb_2_O_5_	Tief-tief	773–873	[11]
T-Nb_2_O_5_	Tief (γ)	873–1073	[12,13]
B-Nb_2_O_5_	Blätter (ζ)	1023–1123	[14]
M-Nb_2_O_5_	Medium (β)	1173–1223	[15]
H-Nb_2_O_5_	High (α)	1273	[16]
N-Nb_2_O_5_	Needles	1103	[17]
P-Nb_2_O_5_	Prisms (η)	1023	[11]
R-Nb_2_O_5_	neutral	-	[18]
ε-Nb_2_O_5_	-	1708	[11]
Nb_2_O_5_-I-high	-	1558	[11]
Nb_2_O_5_-II	-	1153–1223	[11]
The oxI to oxVI Forms of—Nb_2_O_5_	oxidation	1573	[11]

**Table 2:** Structure parameter of various Nb_2_O_5_ structures.

Materials	Crystal Structure	Cell Parameter	Space Group	Ref.
TT-Nb_2_O_5_	Pseudohexagonal	a = b = 3.607 Å/a = b = 3.600 Å c = 3.925 Å/c = 3.919 Å	*P6/mmm* (No. 191)	[12,20]
T-Nb_2_O_5_	Orthorhombic	a = 6.75 Å/a = 6.144 Å b = 29.175 Å/b = 29.194 Å c = 3.930 Å/c = 3.940 Å	*Pbam* (No. 55)	[12,13]
M-Nb_2_O_5_	Tetragonal	a = b = 20.44 Å c = 3.832 Å	*I4/mmm* (No. 139)	[21]
H-Nb_2_O_5_	Monoclinic	a = 21.153 Å/a = 21.163 Å b = 3.8233 Å/b = 3.824 Å c = 19.356 Å/c = 19.355 Å	*P2/m* (No. 10)	[13,21]

## Data Availability

No new data were created or analyzed in this study. Data sharing is not applicable to this article.

## References

[1] Nowak I., Ziolek M. (1999). Niobium Compounds:  Preparation, Characterization, and Application in Heterogeneous Catalysis. Chem. Rev..

[2] Wu J., Xue D. (2011). Localized crystallization: A chemical transformation of Nb_2_O_5_ rod-like arrays into ordered niobate arrays. CrystEngComm.

[3] Zhang B., Deng L., Liebau M., Ren Y., Luo C., Liu B., Zhang S., Gläser R. (2022). Promotion effect of niobium on ceria catalyst for selective catalytic reduction of NO with NH_3_. J. Rare Earths.

[4] Brauer G. (1941). Die Oxyde des Niobs. Anorg. Allg. Chem..

[5] Liu M., Xue D. (2010). Large-scale fabrication of H_2_(H_2_O)Nb_2_O_6_ and Nb_2_O_5_ hollow microspheres. Mater. Res. Bull..

[6] Su K., Liu H., Gao Z., Fornasiero P., Wang F. (2021). Nb_2_O_5_-Based Photocatalysts. Adv. Sci..

[7] Yi T.F., Sari H.M.K., Li X., Wang F., Zhu Y.R., Hu J., Zhang J., Li X. (2021). A review of niobium oxides based nanocomposites for lithium-ion batteries, sodium-ion batteries and supercapacitors. Nano Energy.

[8] Ohzuku T., Sawai K., Hirai T. (1987). Electrochemistry of L-niobium pentoxide a lithium/non-aqueous cell. J. Power Sources.

[9] Ding J., Hu W., Paek E., Mitlin D. (2018). Review of Hybrid Ion Capacitors: From Aqueous to Lithium to Sodium. Chem. Rev..

[10] Dhawan S., Dhawan T., Vedeshwar A.G. (2014). Residual stress induced crystalline to amorphous phase transformation in Nb_2_O_5_ quantum dots. J. Appl. Phys..

[11] Schäfer H., Gruehn R., Schulte F. (1966). The Modifications of Niobium Pentoxide. Angew. Chem. Int. Ed..

[12] Ikeya T., Senna M. (1988). Change in the structure of niobium pentoxide due to mechanical and thermal treatments. J. Non-Cryst. Solids.

[13] Huang H., Kuai H., Ding X., Hu B., Chen Y., Zhou Q., Xiong X. (2023). Single crystal H-Nb_2_O_5_ growing along the [001] crystal direction for ultrafast lithium storage. J. Mater. Chem. A.

[14] Pinto M.B., Soares A.L., Quintão M.C., Duarte H.A., De Abreu H.A. (2018). Unveiling the Structural and Electronic Properties of the B-Nb_2_O_5_ Surfaces and Their Interaction with H_2_O and H_2_O_2_. J. Phys. Chem. C.

[15] Mertin W., Andersson S., Gruehn R. (1970). Über die Kristallstruktur von M-Nb_2_O_5_. J. Solid State Chem..

[16] Kato K. (1976). Structure refinement of H-Nb_2_O_5_. Acta Crystallogr. Sect. B Struct. Crystallogr. Cryst. Chem..

[17] Andersson S. (1967). The Crystal Structure of N-Nb_2_O_5_, prepared in the presence of small amounts of LiF. Z. Anorg. Allg. Chem..

[18] Israelsson M., Kihlborg L. (1970). The crystal structure of monoclinic wolfram vanadium oxide, W_3_V_5_O_20_, an OD structure related to R-Nb_2_O_5_. J. Solid State Chem..

[19] Reznichenko L.A., Akhnazarova V.V., Shilkina L.A., Razumovskaya O.N., Dudkina S.I. (2009). Invar effect in N-Nb_2_O_5_, αht-Nb_2_O_5_, and L-Nb_2_O_5_. Crystallogr. Rep..

[20] Bowman A., Wallace T., Yarnell J., Wenzel R. (1966). The crystal structure of niobium monoxide. Acta Crystallogr..

[21] Park H., Lee D., Song T. (2019). High capacity monoclinic Nb_2_O_5_ and semiconducting NbO_2_ composite as high-power anode material for Li-Ion batteries. J. Power Sources.

[22] Griffith K.J., Forse A.C., Griffin J.M., Grey C.P. (2016). High-rate intercalation without nanostructuring in metastable Nb_2_O_5_ bronze phases. J. Am. Chem. Soc..

[23] Ko E.I., Weissman J.G. (1990). Structures of niobium pentoxide and their implications on chemical behavior. Catal. Today.

[24] Pinto M.B., Soares A.L., Orellana A.M., Duarte H.A., De Abreu H.A. (2017). Structural, Electronic, and Thermodynamic Properties of the T and B Phases of Niobia: First-Principle Calculations. J. Phys. Chem. A.

[25] Zhao Y., Zhou X., Ye L., Chi Edman Tsang S. (2012). Nanostructured Nb_2_O_5_ catalysts. Nano Rev..

[26] Serghiou G.C., Winters R.R., Hammack W.S. (1992). Pressure-induced amorphization and reduction of T-Nb_2_O_5_. Phys. Rev. Lett..

[27] Li L., Deng J., Yu R., Chen J., Wang X., Xing X. (2010). Phase evolution in low-dimensional niobium oxide synthesized by a topochemical method. Inorg. Chem..

[28] Augustyn V., Come J., Lowe M.A., Kim J.W., Taberna P.L., Tolbert S.H., Abruna H.D., Simon P., Dunn B. (2013). High-rate electrochemical energy storage through Li^+^ intercalation pseudocapacitance. Nat. Mater..

[29] Kong L.P., Zhang C.F., Zhang S.M., Wang J.T., Cai R., Lv C.X., Qiao W.M., Ling L.C., Long D.H. (2014). High-power and high-energy asymmetric supercapacitors based on Li^+^-intercalation into a T-Nb_2_O_5_/graphene pseudocapacitive electrode. J. Mater. Chem. A.

[30] Lim E., Jo C., Kim H., Kim M.-H., Mun Y., Chun J., Ye Y., Hwang J., Ha K.-S., Roh K.C. (2015). Facile Synthesis of Nb_2_O_5_@Carbon Core–Shell Nanocrystals with Controlled Crystalline Structure for High-Power Anodes in Hybrid Supercapacitors. ACS Nano.

[31] Liu F.F., Cheng X.L., Xu R., Wu Y., Jiang Y., Yu Y. (2018). Binding Sulfur-Doped Nb_2_O_5_ Hollow Nanospheres on Sulfur-Doped Graphene Networks for Highly Reversible Sodium Storage. Adv. Funct. Mater..

[32] Li N., Zhang F., Tang Y.B. (2018). Hierarchical T-Nb_2_O_5_ nanostructure with hybrid mechanisms of intercalation and pseudocapacitance for potassium storage and high-performance potassium dual-ion batteries. J. Mater. Chem. A.

[33] Wang J.Y., Li G.R., Luo D., Zhao Y., Zhang Y.G., Zhou G.F., Shui L.L., Wang X. (2021). Amorphous-crystalline-heterostructured niobium oxide as two-in-one host matrix for high-performance lithium-sulfur batteries. J. Mater. Chem. A.

[34] Hu Z., He Q., Liu Z., Liu X., Qin M., Wen B., Shi W., Zhao Y., Li Q., Mai L. (2020). Facile formation of tetragonal-Nb_2_O_5_ microspheres for high-rate and stable lithium storage with high areal capacity. Sci. Bull..

[35] Liao J., Tan R., Kuang Z., Cui C., Wei Z., Deng X., Yan Z., Feng Y., Li F., Wang C. (2018). Controlling the morphology, size and phase of Nb_2_O_5_ crystals for high electrochemical performance. Chin. Chem. Lett..

[36] Chen D., Wang J.-H., Chou T.-F., Zhao B., El-Sayed M.A., Liu M. (2017). Unraveling the nature of anomalously fast energy storage in T-Nb_2_O_5_. J. Am. Chem. Soc..

[37] Zhang S., Liu G., Qiao W., Wang J., Ling L. (2020). Oxygen vacancies enhance the lithium ion intercalation pseudocapacitive properties of orthorhombic niobium pentoxide. J. Colloid Interface Sci..

[38] Holtzberg F., Reisman A., Berry M., Berkenblit M. (1957). Chemistry of the Group VB Pentoxides. VI. The Polymorphism of Nb_2_O_5_. J. Am. Chem. Soc..

[39] Shafer M.W., Roy R. (1958). The Polymorphism of Nb_2_O_5_. Z. Kristallogr Cryst. Mater..

[40] Terao N. (1963). Structures des Oxydes de Niobium. Jpn. J. Appl. Phys..

[41] Tamura S. (1972). High-pressure phase research on Nb_2_O_5_. J. Mater. Sci..

[42] Weissman J.G., Ko E.I., Wynblatt P., Howe J.M. (1989). High-resolution electron microscopy and image simulation of TT-,T-, and H-niobia and model silica-supported niobium surface oxides. Chem. Mater..

[43] Košutová T., Horák L., Pleskunov P., Hanuš J., Nikitin D., Kúš P., Cieslar M., Gordeev I., Burazer S., Choukourov A. (2022). Thermally-driven morphogenesis of niobium nanoparticles as witnessed by in-situ X-ray scattering. Mater. Chem. Phys..

[44] Taques Tractz G., Staciaki da Luz F., Regina Masetto Antunes S., do Prado Banczek E., Taras da Cunha M., Rogério Pinto Rodrigues P. (2021). Nb_2_O_5_ synthesis and characterization by Pechini method to the application as electron transport material in a solar device. Sol. Energy.

[45] Schäfer H., Schulte F., Gruehn R. (1964). Neue Nb_2_O_5_-Modifikationen. Angew. Chem..

[46] Uyeda N., Fujiyoshi Y., Ishizuka K. (1984). High resolution imaging and interpretation of regular and irregular structures in N-niobium pentoxide crystal. Ultramicroscopy.

[47] Barnes P., Zuo Y., Dixon K., Hou D., Lee S., Ma Z., Connell J.G., Zhou H., Deng C., Smith K. (2022). Electrochemically induced amorphous-to-rock-salt phase transformation in niobium oxide electrode for Li-ion batteries. Nat. Mater..

[48] Shi C., Xiang K., Zhu Y., Zhou W., Chen X., Chen H. (2017). Box-implanted Nb_2_O_5_ nanorods as superior anode materials in lithium ion batteries. Ceram. Int..

[49] Wang Y., Xin F., Yin X., Song Y., Xiang T., Wang J. (2018). Arginine-Assisted Hydrothermal Synthesis of Urchin-like Nb_2_O_5_ Nanostructures Composed of Nanowires and Their Application in Cyclohexanone Ammoximation. J. Phys. Chem. C.

[50] Zhou Y., Qiu Z., Lü M., Zhang A., Ma Q. (2008). Preparation and characterization of porous Nb_2_O_5_ nanoparticles. Mater. Res. Bull..

[51] Ristić M., Popović S., Musić S. (2004). Sol–gel synthesis and characterization of Nb_2_O_5_ powders. Mater. Lett..

[52] Cheong J.Y., Jung J.-W., Youn D.-Y., Kim C., Yu S., Cho S.-H., Yoon K.R., Kim I.-D. (2017). Mesoporous orthorhombic Nb_2_O_5_ nanofibers as pseudocapacitive electrodes with ultra-stable Li storage characteristics. J. Power Sources.

[53] Yan C., Nikolova L., Dadvand A., Harnagea C., Sarkissian A., Perepichka D.F., Xue D., Rosei F. (2010). Multiple NaNbO_3_/Nb_2_O_5_ heterostructure nanotubes: A new class of ferroelectric/semiconductor nanomaterials. Adv. Mater..

[54] Liu F., Xue D. (2009). Controlled fabrication of Nb_2_O_5_ hollow nanospheres and nanotubes. Modern Phys. Lett. B.

[55] Liu F., Xue D. (2010). Fabrication of Nb_2_O_5_ nanotrees with controlled branching degrees. Phys. Scr..

[56] Yan C., Xue D. (2008). Formation of Nb_2_O_5_ Nanotube Arrays through Phase Transformation. Adv. Mater..

[57] Draper P.H.G., Harvey J. (1963). The structure of anodic films—I. An electron diffraction examination of the products of anodic oxidation on tantalum, niobium and zirconium. Acta Metall..

[58] Arora M.R., Kelly R. (1977). The structure and stoichiometry of anodic films on V, Nb, Ta, Mo and W. J. Mater. Sci..

[59] Aladjem A., Brandon D.G., Yahalom J., Zahavi J. (1970). Electron-beam crystallization of anodic oxide films. Electrochim. Acta.

[60] Habazaki H., Oikawa Y., Fushimi K., Aoki Y., Shimizu K., Skeldon P., Thompson G.E. (2009). Importance of water content in formation of porous anodic niobium oxide films in hot phosphate–glycerol electrolyte. Electrochim. Acta.

[61] Ou J.Z., Rani R.A., Ham M.-H., Field M.R., Zhang Y., Zheng H., Reece P., Zhuiykov S., Sriram S., Bhaskaran M. (2012). Elevated Temperature Anodized Nb_2_O_5_: A Photoanode Material with Exceptionally Large Photoconversion Efficiencies. ACS Nano.

[62] Karlinsey R.L. (2005). Preparation of self-organized niobium oxide microstructures via potentiostatic anodization. Electrochem. Commun..

[63] Karlinsey R.L. (2006). Self-assembled Nb_2_O_5_ microcones with tailored crystallinity. J. Mater. Sci..

[64] Wei W., Lee K., Shaw S., Schmuki P. (2012). Anodic formation of high aspect ratio, self-ordered Nb_2_O_5_ nanotubes. Chem. Commun..

[65] Lee K., Yang Y., Yang M., Schmuki P. (2012). Formation of Highly Ordered Nanochannel Nb Oxide by Self-Organizing Anodization. Chem.–A Eur. J..

[66] Abdul Rani R., Zoolfakar A.S., Subbiah J., Ou J.Z., Kalantar-zadeh K. (2014). Highly ordered anodized Nb_2_O_5_ nanochannels for dye-sensitized solar cells. Electrochem. Commun..

[67] Rani R.A., Zoolfakar A.S., Ou J.Z., Kadir R.A., Nili H., Latham K., Sriram S., Bhaskaran M., Zhuiykov S., Kaner R.B. (2013). Reduced impurity-driven defect states in anodized nanoporous Nb_2_O_5_: The possibility of improving performance of photoanodes. Chem. Commun..

[68] Yao D.D., Rani R.A., O’Mullane A.P., Kalantar-zadeh K., Ou J.Z. (2014). High Performance Electrochromic Devices Based on Anodized Nanoporous Nb_2_O_5_. J. Phys. Chem. C.

[69] Liu X.L., Yuan R.L., Liu Y.S., Zhu S., Lin J., Chen X.F. (2016). Niobium pentoxide nanotube powder for efficient dye-sensitized solar cells. New J. Chem..

[70] Khairir N.S., Rani R.A., Ab Kadir R., Soin N., Abdullah W.F.H., Mamat M.H., Rusop M., Zoolfakar A.S. (2019). Electrical Behavior of a Nanoporous Nb_2_O_5_/Pt Schottky Contact at Elevated Temperatures. J. Electron. Mater..

[71] Pligovka A., Yunin P., Hoha A., Korolyov S., Gorokh G., Skorokhodov E. (2020). Morphology and Structure of Defected Niobium Oxide Nonuniform Arrays Formed by Anodizing Bilayer Al/Nb Systems. Tech. Phys..

[72] Gorokh G.G., Pligovka A.N., Lozovenko A. (2019). Columnar niobium oxide nanostructures: Mechanism of formation, microstructure, and electrophysical properties. Tech. Phys..

[73] Pligovka A., Hoha A., Turavets U., Poznyak A., Zakharau Y. (2021). Formation features, morphology and optical properties of nanostructures via anodizing Al/Nb on Si and glass. Mater. Today Proc..

[74] Pligovka A., Lazavenka A., Zakhlebayeva A. (2018). Electro-physical properties of niobia columnlike nanostructures via the anodizing of Al/Nb layers. Proceedings of the 2018 IEEE 18th International Conference on Nanotechnology (IEEE-NANO).

[75] Pligovka A., Lazavenka A., Turavets U., Hoha A., Salerno M. (2023). Two-Level 3D Column-like Nanofilms with Hexagonally–Packed Tantalum Fabricated via Anodizing of Al/Nb and Al/Ta Layers—A Potential Nano-Optical Biosensor. Materials.

[76] Matsuda A., Kawamura G. (2016). Sol-Gel Nano-/Micropatterning Process. Handbook of Sol-Gel Science and Technology.

[77] Ulrich D.R. (1988). Prospects of sol-gel processes. J. Non-Cryst. Solids.

[78] Hench L.L., West J.K. (1990). The sol-gel process. Chem. Rev..

[79] Nistico R., Scalarone D., Magnacca G. (2017). Sol-gel chemistry, templating and spin-coating deposition: A combined approach to control in a simple way the porosity of inorganic thin films/coatings. Microporous Mesoporous Mater..

[80] Bokov D., Jalil A.T., Chupradit S., Suksatan W., Ansari M.J., Shewael I.H., Valiev G.H., Kianfar E. (2021). Nanomaterial by Sol-Gel Method: Synthesis and Application. Adv. Mater. Sci. Eng..

[81] Niederberger M., Pinna N. (2009). Metal Oxide Nanoparticles in Organic Solvents: Synthesis, Formation, Assembly and Application.

[82] Alquier C., Vandenborre M.T., Henry M. (1986). Synthesis of niobium pentoxide gels. J. Non-Cryst. Solids.

[83] Schmitt M., Heusing S., Aegerter M.A., Pawlicka A., Avellaneda C. (1998). Electrochromic properties of Nb_2_O_5_ sol-gel coatings. Sol. Energy Mater. Sol. Cells.

[84] Melo L., Avellaneda C.O., Caram R., Sichieri E., Pawlicka A. (2002). Electrochromic Properties of Sol-gel Coating of Nb_2_O_5_ and Nb_2_O_5_: Li^+^. Mater. Res..

[85] Xu Z.Y., Shen X.M., Wu X.R., Cao F.B., Li L.S. (2021). Effect of crystallization degree on optical characteristics of Nb_2_O_5_ by sol-gel process. Mod. Phys. Lett. B.

[86] Pu J., Shen Z.H., Zhong C.L., Zhou Q.W., Liu J.Y., Zhu J., Zhang H.G. (2020). Electrodeposition Technologies for Li-Based Batteries: New Frontiers of Energy Storage. Adv. Mater..

[87] Jang J.-H., Kim T.-Y., Kim N.-J., Lee C.-H., Park E.-M., Park C., Suh S.-J. (2011). Preparation and characterization of Nb_2_O_5_–Al_2_O_3_ composite oxide formed by cathodic electroplating and anodizing. Mater. Sci. Eng. B.

[88] Jha G., Tran T., Qiao S.P., Ziegler J.M., Ogata A.F., Dai S., Xu M.J., Le Thai M., Chandran G.T., Pan X.Q. (2018). Electrophoretic Deposition of Mesoporous Niobium(V)Oxide Nanoscopic Films. Chem. Mater..

[89] RobertáLee G. (1996). Studies on the electrochemical deposition of niobium oxide. J. Mater. Chem..

[90] Zhitomirsky I. (1999). Electrolytic deposition of oxide films in the presence of hydrogen peroxide. J. Eur. Ceram. Soc..

[91] Zhitomirsky I. (1998). Electrolytic deposition of niobium oxide films. Mater. Lett..

[92] Kamada K., Mukai M., Matsumoto Y. (2004). Anodic dissolution of tantalum and niobium in acetone solvent with halogen additives for electrochemical synthesis of Ta_2_O_5_ and Nb_2_O_5_ thin films. Electrochim. Acta.

[93] Zhao G.Y., Ye C., Zhang L., Li C.L., Sun K.N. (2017). T-Nb_2_O_5_ quantum dots prepared by electrodeposition for fast Li ion intercalation/deintercalation. Nanotechnology.

[94] Yang D. (2011). Nanocomposite Films for Gas Sensing.

[95] Yap Y. (2012). Physical Vapor Deposition, Encyclopedia of Nanotechnology: Springer Reference.

[96] Moreto J., Gelamo R., Nascimento J., Taryba M., Fernandes J. (2021). Improving the corrosion protection of 2524-T3-Al alloy through reactive sputtering Nb_2_O_5_ coatings. Appl. Surf. Sci..

[97] Çetinörgü-Goldenberg E., Klemberg-Sapieha J.-E., Martinu L. (2012). Effect of postdeposition annealing on the structure, composition, and the mechanical and optical characteristics of niobium and tantalum oxide films. Appl. Opt..

[98] Tien C.-L. (2010). Effect of sputtering anisotropic ejection on the optical properties and residual stress of Nb_2_O_5_ thin films. Appl. Surf. Sci..

[99] Reichman B., Bard A.J. (1980). Electrochromism at niobium pentoxide electrodes in aqueous and acetonitrile solutions. J. Electrochem. Soc..

[100] Vinnichenko M., Rogozin A., Grambole D., Munnik F., Kolitsch A., Möller W., Stenzel O., Wilbrandt S., Chuvilin A., Kaiser U. (2009). Highly dense amorphous Nb_2_O_5_ films with closed nanosized pores. Appl. Phys. Lett..

[101] Pillis M.F., Geribola G.A., Scheidt G., de Araújo E.G., de Oliveira M.C.L., Antunes R.A. (2016). Corrosion of thin, magnetron sputtered Nb_2_O_5_ films. Corros. Sci..

[102] Coşkun Ö.D., Demirela S. (2013). The optical and structural properties of amorphous Nb_2_O_5_ thin films prepared by RF magnetron sputtering. Appl. Surf. Sci..

[103] Chen K.-N., Hsu C.-M., Liu J., Liou Y.-C., Yang C.-F. (2016). Investigation of antireflection Nb_2_O_5_ thin films by the sputtering method under different deposition parameters. Micromachines.

[104] Al-Baradi A.M., El-Nahass M., Hassanien A., Atta A., Alqahtani M.S., Aldawsari A.O. (2018). Influence of RF sputtering power on structural and optical properties of Nb_2_O_5_ thin films. Optik.

[105] Dhar A., Alford T. (2012). Optimization of Nb_2_O_5_/Ag/Nb_2_O_5_ multilayers as transparent composite electrode on flexible substrate with high figure of merit. J. Appl. Phys..

[106] Venkataraj S., Drese R., Kappertz O., Jayavel R., Wuttig M. (2001). Characterization of niobium oxide films prepared by reactive DC magnetron sputtering. Phys. Status Solidi A.

[107] Bussamara R., Eberhardt D., Feil A., Migowski P., Wender H., de Moraes D.P., Machado G., Papaléo R.M., Teixeira S.R., Dupont J. (2013). Sputtering deposition of magnetic Ni nanoparticles directly onto an enzyme surface: A novel method to obtain a magnetic biocatalyst. Chem. Commun..

[108] Kuzminykh Y., Dabirian A., Reinke M., Hoffmann P. (2013). High vacuum chemical vapour deposition of oxides: A review of technique development and precursor selection. Surf. Coat. Technol..

[109] Jung S.-C., Imaishi N., Park H.-C. (1995). Reaction engineering modeling of low-pressure metalorganic chemical vapor deposition of Nb_2_O_5_ thin film. Jpn. J. Appl. Phys..

[110] Breiland W.G., Coltrin M.E., Creighton J.R., Hou H.Q., Moffat H.K., Tsao J.Y. (1999). Organometallic vapor phase epitaxy (OMVPE). Mater. Sci. Eng. R Rep..

[111] Mueller N.S., Morfa A.J., Abou-Ras D., Oddone V., Ciuk T., Giersig M. (2014). Growing graphene on polycrystalline copper foils by ultra-high vacuum chemical vapor deposition. Carbon.

[112] Baum T.H., Larson C.E., Jackson R.L. (1989). Laser-induced chemical vapor deposition of aluminum. Appl. Phys. Lett..

[113] O’Neill S.A., Parkin I.P., Clark R.J., Mills A., Elliott N. (2003). Atmospheric pressure chemical vapour deposition of thin films of Nb_2_O_5_ on glass. J. Mater. Chem..

[114] de Oliveira A.F., da Silveira C.B., de Campos S.D., de Campos E.A., Carasek E. (2005). Niobium (V) oxide coated on thin glass–ceramic rod as a solid phase microextraction fiber. Talanta.

[115] da Silveira C., de Oliveira A., de Campos S., de Campos E., Fraporti A. (2012). Nb_2_O_5_ coating of glass fibres applied by chemical vapour deposition. Surf. Eng..

[116] Kovendhan M., Joseph D.P., Manimuthu P., Ganesan S., Sambasivam S., Maruthamuthu P., Suthanthiraraj S.A., Venkateswaran C., Mohan R. (2011). Spray deposited Nb_2_O_5_ thin film electrodes for fabrication of dye sensitized solar cells. Trans. Indian Inst. Met..

[117] Xia J., Masaki N., Jiang K., Yanagida S. (2007). Fabrication and characterization of thin Nb_2_O_5_ blocking layers for ionic liquid-based dye-sensitized solar cells. J. Photochem. Photobiol. A.

[118] Wang Z., Hu Y., Wang W., Zhang X., Wang B., Tian H., Wang Y., Guan J., Gu H. (2012). Fast and highly-sensitive hydrogen sensing of Nb_2_O_5_ nanowires at room temperature. Int. J. Hydrogen Energy.

[119] Tamang R., Varghese B., Mhaisalkar S.G., Tok E.S., Sow C.H. (2011). Probing the photoresponse of individual Nb_2_O_5_ nanowires with global and localized laser beam irradiation. Nanotechnology.

[120] Nico C., Rino L., Matos M., Monteiro R., Costa F.M., Monteiro T., Graça M.P. (2013). NbO/Nb_2_O_5_ core–shells by thermal oxidation. J. Eur. Ceram. Soc..

[121] Lim J.H., Choi J. (2009). Formation of niobium oxide nanowires by thermal oxidation. J. Ind. Eng. Chem..

[122] De Sá A., Rangel C., Skeldon P., Thompson G. (2006). Semiconductive properties of anodic niobium oxides. Port. Electrochim. Acta.

[123] Luo H., Song W., Hoertz P.G., Hanson K., Ghosh R., Rangan S., Brennaman M.K., Concepcion J.J., Binstead R.A., Bartynski R.A. (2013). A sensitized Nb_2_O_5_ photoanode for hydrogen production in a dye-sensitized photoelectrosynthesis cell. Chem. Mater..

[124] Graça M.P.F., Meireles A., Nico C., Valente M.A. (2013). Nb_2_O_5_ nanosize powders prepared by sol–gel–Structure, morphology and dielectric properties. J. Alloys Compd..

[125] Jiayu D., Zhefei W., Zhijie G., Yuan X., Lixi W., ZHANG Q. (2011). Optimization on dielectric properties of Y_2_Ti_2_O_7_ ceramics with Bi_2_O_3_-Nd_2_O_3_-Nb_2_O_5_ co-doping. J. Rare Earths.

[126] Clima S., Pourtois G., Van Elshocht S., De Gendt S., Heyns M.M., Wouters D.J., Kittl J.A. (2009). Dielectric response of Ta_2_O_5_, NbTaO_5_ and Nb_2_O_5_ from first-principles investigations. ECS Trans..

[127] García H., Castán H., Perez E., Dueñas S., Bailón L., Blanquart T., Niinistö J., Kukli K., Ritala M., Leskelä M. (2013). Influence of growth and annealing temperatures on the electrical properties of Nb_2_O_5_-based MIM capacitors. Semicond. Sci. Technol..

[128] Soares M., Leite S., Nico C., Peres M., Fernandes A., Graça M., Matos M., Monteiro R., Monteiro T., Costa F. (2011). Effect of processing method on physical properties of Nb_2_O_5_. J. Eur. Ceram. Soc..

[129] Greener E., Hirthe W. (1962). Electrical Conductivity of Nonstoichiometric α-Nb_2_O_5_. J. Electrochem. Soc..

[130] Nanda G., Awin E.W., Gasyak T., Koroleva E., Filimonov A., Vakhrushev S., Sujith R., Kumar R. (2020). Temperature dependent conductivity and broadband dielectric response of precursor-derived Nb_2_O_5_. Ceram. Int..

[131] Lazarova K., Vasileva M., Marinov G., Babeva T. (2014). Optical characterization of sol–gel derived Nb_2_O_5_ thin films. Opt. Laser Technol..

[132] Abood M., Wahid M., Saimon J., Salim E. (2018). Physical properties of Nb_2_O_5_ thin films prepared at 12M ammonium concentration. Int. J. Nanoelectron. Mater..

[133] Sun S., Mottram L., Hyatt N.C. (2021). On the existence of the compound “Ce_3_NbO_7+δ_” prepared under air atmosphere. J. Rare Earths.

[134] Fakhri M.A., Al-Douri Y., Hashim U., Salim E.T. (2015). Optical investigations of photonics lithium niobate. Sol. Energy.

[135] Le Viet A., Jose R., Reddy M., Chowdari B., Ramakrishna S. (2010). Nb_2_O_5_ photoelectrodes for dye-sensitized solar cells: Choice of the polymorph. J. Phys. Chem. C.

[136] Abe S. (2012). Formation of Nb_2_O_5_ matrix and Vis-NIR absorption in Nb-Ge-O thin film. Nanoscale Res. Lett..

[137] Liu J., Xue D., Li K. (2011). Single-crystalline nanoporous Nb_2_O_5_ nanotubes. Nanoscale Res. Lett..

[138] Brayner R., Bozon-Verduraz F. (2003). Niobium pentoxide prepared by soft chemical routes: Morphology, structure, defects and quantum size effect. Phys. Chem. Chem. Phys..

[139] Agarwal G., Reddy G. (2005). Study of surface morphology and optical properties of Nb_2_O_5_ thin films with annealing. J. Mater. Sci. Mater. Electron..

[140] Aegerter M.A., Schmitt M., Guo Y. (2002). Sol-gel niobium pentoxide coatings: Applications to photovoltaic energy conversion and electrochromism. Int. J. Photoenergy.

[141] Xiao X., Dong G., Xu C., He H., Qi H., Fan Z., Shao J. (2008). Structure and optical properties of Nb_2_O_5_ sculptured thin films by glancing angle deposition. Appl. Surf. Sci..

[142] Shcherbina O., Palatnikov M., Efremov V. (2012). Mechanical properties of Nb_2_O_5_ and Ta_2_O_5_ prepared by different procedures. Inorg. Mater..

[143] Rani R.A., Zoolfakar A.S., O’Mullane A.P., Austin M.W., Kalantar-Zadeh K. (2014). Thin films and nanostructures of niobium pentoxide: Fundamental properties, synthesis methods and applications. J. Mater. Chem. A.

[144] Cavers H., Molaiyan P., Abdollahifar M., Lassi U., Kwade A. (2022). Perspectives on improving the safety and sustainability of high voltage lithium-ion batteries through the electrolyte and separator region. Adv. Energy Mater..

[145] Armand M., Tarascon J.-M. (2008). Building better batteries. Nature.

[146] Simon P., Gogotsi Y. (2008). Materials for electrochemical capacitors. Nat. Mater..

[147] Zhang H., Yang Y., Ren D., Wang L., He X. (2021). Graphite as anode materials: Fundamental mechanism, recent progress and advances. Energy Storage Mater..

[148] Ren J., Ren R.P., Lv Y.K. (2018). A New Anode for Lithium-Ion Batteries Based on Single-Walled Carbon Nanotubes and Graphene: Improved Performance through a Binary Network Design. Chem.–A Asian J..

[149] Abdollahifar M., Molaiyan P., Perovic M., Kwade A. (2022). Insights into Enhancing Electrochemical Performance of Li-Ion Battery Anodes via Polymer Coating. Energies.

[150] Molaiyan P., Dos Reis G.S., Karuppiah D., Subramaniyam C.M., Garcia-Alvarado F., Lassi U. (2023). Recent progress in biomass-derived carbon materials for Li-ion and Na-ion batteries—A review. Batteries.

[151] Välikangas J., Laine P., Hu T., Tynjälä P., Selent M., Molaiyan P., Jürgen K., Lassi U. (2023). Effect of Secondary Heat Treatment after a Washing on the Electrochemical Performance of Co-Free LiNi_0.975_Al_0.025_O_2_ Cathodes for Li-Ion Batteries. Small.

[152] Yuan C., Wu H.B., Xie Y., Lou X.W. (2014). Mixed transition-metal oxides: Design, synthesis, and energy-related applications. Angew. Chem. Int. Ed..

[153] Deng Q., Fu Y., Zhu C., Yu Y. (2019). Niobium-based oxides toward advanced electrochemical energy storage: Recent advances and challenges. Small.

[154] Zhou Z., Lou S., Cheng X., Cui B., Si W., Ding F., Ma Y., Zuo P., Du C., Wang J. (2020). Superior Electrochemical Performance of WNb_2_O_8_ Nanorods Triggered by Ultra-Efficient Li^+^ Diffusion. ChemistrySelect.

[155] Lin J., Yuan Y., Su Q., Pan A., Dinesh S., Peng C., Cao G., Liang S. (2018). Facile synthesis of Nb_2_O_5_/carbon nanocomposites as advanced anode materials for lithium-ion batteries. Electrochim. Acta.

[156] Shen F., Sun Z., He Q., Sun J., Kaner R.B., Shao Y. (2021). Niobium pentoxide based materials for high rate rechargeable electrochemical energy storage. Mater. Horiz..

[157] Griffith K.J., Wiaderek K.M., Cibin G., Marbella L.E., Grey C.P. (2018). Niobium tungsten oxides for high-rate lithium-ion energy storage. Nature.

[158] Etacheri V., Marom R., Elazari R., Salitra G., Aurbach D. (2011). Challenges in the development of advanced Li-ion batteries: A review. Energy Environ. Sci..

[159] Reddy M.V., Subba Rao G.V., Chowdari B.V.R. (2013). Metal Oxides and Oxysalts as Anode Materials for Li Ion Batteries. Chem. Rev..

[160] Ding H., Song Z., Zhang H., Zhang H., Li X. (2020). Niobium-based oxide anodes toward fast and safe energy storage: A review. Mater. Today Nano.

[161] Yang M., Li S., Huang J. (2021). Three-Dimensional Cross-Linked Nb_2_O_5_ Polymorphs Derived from Cellulose Substances: Insights into the Mechanisms of Lithium Storage. ACS Appl. Mater. Interfaces.

[162] Li M., Lu J., Chen Z., Amine K. (2018). 30 years of lithium-ion batteries. Adv. Mater..

[163] Reichman B., Bard A.J. (1981). The Application of Nb_2_O_5_ as a Cathode in Nonaqueous Lithium Cells. J. Electrochem. Soc..

[164] Kumagai N., Tanno K., Nakajima T., Watanabe N. (1983). Structural changes of Nb_2_O_5_ and V_2_O_5_ as rechargeable cathodes for lithium battery. Electrochim. Acta.

[165] Kodama R., Terada Y., Nakai I., Komaba S., Kumagai N. (2006). Electrochemical and in situ XAFS-XRD investigation of Nb_2_O_5_ for rechargeable lithium batteries. J. Electrochem. Soc..

[166] Han J.-T., Huang Y.-H., Goodenough J.B. (2011). New anode framework for rechargeable lithium batteries. Chem. Mater..

[167] Nakazawa H., Sano K., Abe T., Baba M., Kumagai N. (2007). Charge–discharge characteristics of all-solid-state thin-filmed lithium-ion batteries using amorphous Nb_2_O_5_ negative electrodes. J. Power Sources.

[168] Kumagai N., Koishikawa Y., Komaba S., Koshiba N. (1999). Thermodynamics and Kinetics of Lithium Intercalation into Nb_2_O_5_ Electrodes for a 2 V Rechargeable Lithium Battery. J. Electrochem. Soc..

[169] Come J., Augustyn V., Kim J.W., Rozier P., Taberna P.-L., Gogotsi P., Long J.W., Dunn B., Simon P. (2014). Electrochemical kinetics of nanostructured Nb_2_O_5_ electrodes. J. Electrochem. Soc..

[170] Liu C.-P., Zhou F., Ozolins V. (2012). First principles study for lithium intercalation and diffusion behavior in orthorhombic Nb_2_O_5_ electrochemical supercapacitor. APS March Meeting Abstracts.

[171] Kim J.W., Augustyn V., Dunn B. (2012). The effect of crystallinity on the rapid pseudocapacitive response of Nb_2_O_5_. Adv. Energy Mater..

[172] Song Z., Li H., Liu W., Zhang H., Yan J., Tang Y., Huang J., Zhang H., Li X. (2020). Ultrafast and stable Li-(de) intercalation in a large single crystal H-Nb_2_O_5_ anode via optimizing the homogeneity of electron and ion transport. Adv. Mater..

[173] Lübke M., Sumboja A., Johnson I.D., Brett D.J.L., Shearing P.R., Liu Z., Darr J.A. (2016). High power nano-Nb_2_O_5_ negative electrodes for lithium-ion batteries. Electrochim. Acta.

[174] Li S., Xu Q., Uchaker E., Cao X., Cao G. (2016). Comparison of amorphous, pseudohexagonal and orthorhombic Nb_2_O_5_ for high-rate lithium ion insertion. CrystEngComm.

[175] Liu Z., Dong W., Wang J., Dong C., Lin Y., Chen I.-W., Huang F. (2020). Orthorhombic Nb_2_O_5−x_ for durable high-rate anode of Li-ion batteries. iScience.

[176] Chen H., Zhang H., Wu Y., Zhang T., Guo Y., Zhang Q., Zeng Y., Lu J. (2018). Nanostructured Nb_2_O_5_ cathode for high-performance lithium-ion battery with Super-P and graphene compound conductive agents. J. Electroanal. Chem..

[177] Ye R., Ohta K., Baba M. (2016). Electrochemical properties of amorphous Nb_2_O_5_ thin film and its application to rechargeable thin film lithium ion batteries. ECS Trans..

[178] Liu M., Yan C., Zhang Y. (2015). Fabrication of Nb_2_O_5_ nanosheets for high-rate lithium ion storage applications. Sci. Rep..

[179] Uchida S., Zettsu N., Hirata K., Kami K., Teshima K. (2016). High-voltage capabilities of ultra-thin Nb_2_O_5_ nanosheet coated LiNi_1/3_Co_1/3_Mn_1/3_O_2_ cathodes. RSC Adv..

[180] Liu X., Liu G., Chen H., Ma J., Zhang R. (2017). Facile synthesis of Nb_2_O_5_ nanobelts assembled from nanorods and their applications in lithium ion batteries. J. Phys. Chem. Solids.

[181] Huang C., Fu J., Song H., Li X., Peng X., Gao B., Zhang X., Chu P.K. (2016). General fabrication of mesoporous Nb_2_O_5_ nanobelts for lithium ion battery anodes. RSC Adv..

[182] Wei M., Wei K., Ichihara M., Zhou H. (2008). Nb_2_O_5_ nanobelts: A lithium intercalation host with large capacity and high rate capability. Electrochem. Commun..

[183] Sun Y.-G., Piao J.-Y., Hu L.-L., Bin D.-S., Lin X.-J., Duan S.-Y., Cao A.-M., Wan L.-J. (2018). Controlling the reaction of nanoparticles for hollow metal oxide nanostructures. J. Am. Chem. Soc..

[184] Lu H., Xiang K., Bai N., Zhou W., Wang S., Chen H. (2016). Urchin-shaped Nb_2_O_5_ microspheres synthesized by the facile hydrothermal method and their lithium storage performance. Mater. Lett..

[185] Liu S., Zhou J., Cai Z., Fang G., Pan A., Liang S. (2016). Nb_2_O_5_ microstructures: A high-performance anode for lithium ion batteries. Nanotechnology.

[186] Liu X., Liu G., Liu Y., Sun R., Ma J., Guo J., Hu M. (2017). Urchin-like hierarchical H-Nb_2_O_5_ microspheres: Synthesis, formation mechanism and their applications in lithium ion batteries. Dalton Trans..

[187] Liu G., Jin B., Bao K., Xie H., Guo J., Ji X., Zhang R., Jiang Q. (2017). Facile synthesis of porous Nb_2_O_5_ microspheres as anodes for lithium-ion batteries. Int. J. Hydrogen Energy.

[188] Lou S., Cheng X., Wang L., Gao J., Li Q., Ma Y., Gao Y., Zuo P., Du C., Yin G. (2017). High-rate capability of three-dimensionally ordered macroporous T-Nb_2_O_5_ through Li^+^ intercalation pseudocapacitance. J. Power Sources.

[189] Li S., Schmidt C.N., Xu Q., Cao X., Cao G. (2016). Macroporous nanostructured Nb_2_O_5_ with surface Nb4+ for enhanced lithium ion storage properties. ChemNanoMat.

[190] Chen J., Wang H., Zhang X., Liu B., Xu L., Zhang Z., Zhang Y. (2018). 2D ultrathin nanosheet-assembled Nb_2_O_5_ microflowers for lithium ion batteries. Mater. Lett..

[191] Li X., Sun X., Hu X., Fan F., Cai S., Zheng C., Stucky G.D. (2020). Review on comprehending and enhancing the initial Coulombic efficiency of anode materials in lithium-ion/sodium-ion batteries. Nano Energy.

[192] Han X., Meng Q., Wan X., Sun B.Y., Zhang Y., Shen B.C., Gao J.L., Ma Y.L., Zuo P.J., Lou S.F. (2021). Intercalation pseudocapacitive electrochemistry of Nb-based oxides for fast charging of lithium-ion batteries. Nano Energy.

[193] Yan L., Rui X., Chen G., Xu W., Zou G., Luo H. (2016). Recent advances in nanostructured Nb-based oxides for electrochemical energy storage. Nanoscale.

[194] Lubimtsev A.A., Kent P.R., Sumpter B.G., Ganesh P. (2013). Understanding the origin of high-rate intercalation pseudocapacitance in Nb_2_O_5_ crystals. J. Mater. Chem. A.

[195] Chen P.-C., Shen G., Shi Y., Chen H., Zhou C. (2010). Preparation and characterization of flexible asymmetric supercapacitors based on transition-metal-oxide nanowire/single-walled carbon nanotube hybrid thin-film electrodes. ACS Nano.

[196] Lim E., Kim H., Jo C., Chun J., Ku K., Kim S., Lee H.I., Nam I.-S., Yoon S., Kang K. (2014). Advanced hybrid supercapacitor based on a mesoporous niobium pentoxide/carbon as high-performance anode. ACS Nano.

[197] Wang X., Yan C., Yan J., Sumboja A., Lee P.S. (2015). Orthorhombic niobium oxide nanowires for next generation hybrid supercapacitor device. Nano Energy.

[198] Wang X., Li G., Chen Z., Augustyn V., Ma X., Wang G., Dunn B., Lu Y. (2011). High-performance supercapacitors based on nanocomposites of Nb_2_O_5_ nanocrystals and carbon nanotubes. Adv. Energy Mater..

[199] Zhang S., Wu J., Wang J., Qiao W., Long D., Ling L. (2018). Constructing T-Nb_2_O_5_@Carbon hollow core-shell nanostructures for high-rate hybrid supercapacitor. J. Power Sources.

[200] Liu S., Zhou J., Cai Z., Fang G., Cai Y., Pan A., Liang S. (2016). Nb_2_O_5_ quantum dots embedded in MOF derived nitrogen-doped porous carbon for advanced hybrid supercapacitor applications. J. Mater. Chem. A.

[201] Ding Y., Zeng M., Fu L. (2019). Low-temperature synthesis of sp2 carbon nanomaterials. Sci. Bull..

[202] Wang X., Li G., Tjandra R., Fan X., Xiao X., Yu A. (2015). Fast lithium-ion storage of Nb_2_O_5_ nanocrystals in situ grown on carbon nanotubes for high-performance asymmetric supercapacitors. RSC Adv..

[203] Ma Y., Han J., Wang M., Chen X., Jia S. (2018). Electrophoretic deposition of graphene-based materials: A review of materials and their applications. J. Mater..

[204] Chen Z., Li H., Lu X., Wu L., Jiang J., Jiang S., Wang J., Dou H., Zhang X. (2018). Nitrogenated Urchin-like Nb_2_O_5_ Microspheres with Extraordinary Pseudocapacitive Properties for Lithium-Ion Capacitors. ChemElectroChem.

[205] Wang J., Li H., Shen L., Dong S., Zhang X. (2016). Nb_2_O_5_ nanoparticles encapsulated in ordered mesoporous carbon matrix as advanced anode materials for Li ion capacitors. RSC Adv..

[206] Zheng J., Cygan P., Jow T. (1995). Hydrous ruthenium oxide as an electrode material for electrochemical capacitors. J. Electrochem. Soc..

[207] Arunkumar P., Ashish A.G., Babu B., Sarang S., Suresh A., Sharma C.H., Thalakulam M., Shaijumon M.M. (2015). Nb_2_O_5_/graphene nanocomposites for electrochemical energy storage. RSC Adv..

[208] Zhang C.J., Maloney R., Lukatskaya M.R., Beidaghi M., Dyatkin B., Perre E., Long D., Qiao W., Dunn B., Gogotsi Y. (2015). Synthesis and electrochemical properties of niobium pentoxide deposited on layered carbide-derived carbon. J. Power Sources.

